# Experimental study on fracture characteristics of rock-like material with prefabricated cracks under compression shear

**DOI:** 10.1038/s41598-022-06712-8

**Published:** 2022-02-18

**Authors:** Shuang-Xi Yuan, Tong Jiang, Jia-Hua Lei, Cheng-Hao Cui

**Affiliations:** College of Geosciences and Engineering, North China University of Water Resources and Electric Power, Zhengzhou, 450045 China

**Keywords:** Civil engineering, Petrology

## Abstract

In order to understand the effects of different patterns of prefabricated fractures and grain size compositions on the fracture characteristics, acoustic emission characteristics and mechanical properties of the rock masses. We conducted compression shear experiments on square rock masses with different modes of prefabricated fractures and different grain size compositions. The experimental results showed that five fracture patterns were produced in specimens with different fracture patterns. The first fracture specimen, the fourth fracture specimen and the fifth fracture specimen were all brittle fractures. The other four specimens were not brittle fractures. The fracture patterns, fracture processes and mechanical characteristics of the different fracture pattern rock masses were revealed. The lowest peak shear stresses were found in specimens consisting of two grain size ranges and the highest peak shear stresses were found in specimens consisting of three grain size ranges. The highest shear displacements corresponding to the peak shear stresses were found in the specimens consisting of three particle size ranges. The effect of different grain size compositions on the peak shear stress and its corresponding shear displacement of the rock mass was revealed. Specimens consisting of one grain size range produced significant fracture and acoustic emission prior to the peak shear stress. The acoustic emission was jumped after the main fracture was formed. The specimen consisting of two grain size ranges produced fractures and strong acoustic emission characteristics after the peak shear stress. Thereafter, fracture reappeared and the acoustic emission signature increased again. As the specimen entered the residual strength phase, the acoustic emission was jumpy. Specimens consisting of three grain size ranges were brittle fractures with weak acoustic emission characteristics after the main fracture has formed. The cumulative energy of shear acoustic emission was the highest for a rock mass consisting of three grain size ranges. The rock mass consisting of three grain size ranges was also the strongest and most difficult to fracture because the grains were more fully embedded in each other.

## Introduction

Fissures are commonly found in rock masses. There are significant interactions between fractures and the rock mass and between pre-existing fractures and fractures. These interactions greatly affect the mechanical properties, fracture characteristics and acoustic emission properties of the rock mass.

Shen found that pre-existing joints propagate as tensile cracks, shear cracks and mixed-mode cracks by conducting an indoor test study on plaster specimens^1^. Bobet and Einstein identified five failure modes in plaster specimens with parallel defects from uniaxial compression tests on plaster specimens with parallel defects^2^. By investigating the coalescence mode between two non-parallel joints, Lee and Jeon found that the joints coalesced mainly through tensile cracks or tensile and shear cracks^3^. To understand how joint geometry parameters affect the deformation modulus, compressive strength and damage mode of rock masses, Bahaaddini et al.^4^ developed a particle flow model of rock masses. To understand the relationship between real-time fracture coalescence processes and axial stress-time behaviour in red sandstones containing two non-parallel fractures, Yang et al.^5,6^ used photographic monitoring and acoustic emission monitoring techniques. By investigating the cohesive behaviour of non-parallel cracks, Zhang et al.^7^ found five types of association between two types of cracks: tensile crack coupling, tensile crack coupling with shear coalescence at the tip, shear crack coupling, mixed coupling and indirect crack coupling. Wong and Chau^8^ found nine coalescence modes for this gypsum by uniaxial compression testing of a gypsum specimen with two defects. By examining the results of uniaxial compression tests on Diastone and Yeosan^9^ marble, Park found that there were seven crack coalescence modes.

In order to understand the peak uniaxial compressive strength and damage modes of ubiquitous-joint rock specimens, Cao et al.^10^ used similar material tests and numerical simulations. They found four damage modes for ubiquitous-joint specimens. To understand how joint inclination and joint connectivity affect the compressive strength and stress–strain curves of rock masses in non-persistent open joints, Chen et al.^11^ carried out uniaxial compression tests on gypsum specimens. Ghazvinian et al.^12^ used the Particle Flow Code 2D (PFC2D) to simulate the shear behaviour of rock-like material samples containing planar non-persistent joints. The damage patterns and mechanisms of the samples were understood and the effect of rock bridges on fracture strength and fracture morphology was understood. Sarfarazi et al.^13^ investigated the effect of joint overlap on the complete damage behaviour of rock bridges by means of the particle flow code 2D (PFC2D). The damage modes and mechanisms were understood. The effect of overlapping joints on damage strength and rupture morphology was understood. Fan et al.^14^ investigated the damage behaviour of fractured rock masses under direct shear loading through physical tests and numerical simulations. They obtained an understanding of the fracture mode and mechanical characteristics of the rock mass. Cao et al.^15^ investigated the peak shear strength and damage process of specimens with multiple joints by laboratory tests and particle flow code (PFC2D). They understood the damage pattern of the specimens and the factors influencing the failure mode and peak strength of the specimens. Wang et al.^16^ carried out compression shear tests on parallel joint specimens in order to investigate the effect of joints on the damage mode, peak shear strength and shear stress–strain curve of the rock mass. They understood the effect of different joints on the fracture mode. They have understood the effect of different joint spacing on the mechanical characteristics of rupture and acoustic emission. Xia et al.^17^ used a modified method of particle flow code to develop a numerical model to investigate the effect of bedding geometry on the direct shear strength properties and fracture mode of transversely isotropic rocks. They understood the effect of the magnitude of the bedding plane dip angle on the fracture mode of the rock. Cen et al.^18^ carried out tensile-shear tests on cubic rock specimens to test the direct shear behaviour of sandstone under different conditions of constant normal tensile stress. They understood the effect of the magnitude of the normal force on fracture surface roughness, total normal deformation and shear strength. Yang et al.^19^ investigated the effects of normal stress and joint persistence on the mechanical behaviour of granite containing discontinuous joints by direct shear tests. They understood the fracture mode of the rock. They understood the pattern of influence of joint persistence and normal force on the fracture mode and shear strength.

Lin et al.^20^ conducted compression-shear tests on nodular rock specimens containing round holes. They revealed the effect of joint inclination on peak loads and crack extension. Luo et al.^21^ carried out compression-shear tests on rock-like specimens with three fissures under four filling conditions: unfilled, gypsum-filled, cement-filled and resin-filled. They revealed the effects of the three fillings on the mechanical characteristics and damage modes of the fractured specimens. Wang et al.^22^ carried out a compression-shear study on self-compacting concrete. They revealed the failure modes and mechanical characteristics of self-compacting concrete. The composition and variation of the shear strength were revealed. Wang et al.^23^ carried out experimental direct shear tests on layered cores of granite rocks. They revealed the effects of normal stress and joint orientation and assessed the anisotropy and directionality of shear strength. Ma et al.^24^ revealed the effect of rock particle gradation and compression ratio on the shear strength properties of regenerated rock structure (RRMS) formed by confined compression of fractured soft rock. They revealed the effect of particle gradation on compaction and surface smoothness. The effects of internal friction angle and internal friction angle of particle grading were revealed. Through the three-point bending (TPB) tests on concrete beams with different sizes, Carpinteri et al.^25^ understood the type of cracks can be identified according to the AE parameters. They understood the different change characteristics of the fracture energy and the acoustic emission energy of the fracture surface. Carpinteri et al.^26^ carried out uniaxial compression test on concrete, rocks-limestone, marble and granite-specimens, the post-peak response of different samples is obtained: from normal softening to catastrophic rebound. They understood acoustic emission parameters can identify the type of crack.

The damage mode, coalescence mode, mechanical properties and stress–strain characteristics of rock masses with prefabricated fractures under uniaxial compression, triaxial compression and compression shear have been studied extensively by previous authors with fruitful results. The coalescence process and the mode of association between two fractures in a rock mass have also been extensively studied with fruitful results.

Thanks to their results, we have gained a better understanding of how fractures in the rock mass extended and how they were connected to each other. We have gained a better understanding of the effects of fractures on the fracture patterns, damage patterns and mechanical properties of rock masses.

However, relatively little research has been carried out on the fracture pattern, mechanical properties and acoustic emission characteristics of end-fractured square rock masses with different fracture patterns and different grain size compositions under compression-shear conditions. In this paper, we investigated the fracture morphology, mechanical properties and acoustic emission characteristics of rock masses with different fracture patterns and different grain size compositions under compression shear conditions.

## Test procedure

### Sample preparation

**Figure 1:**
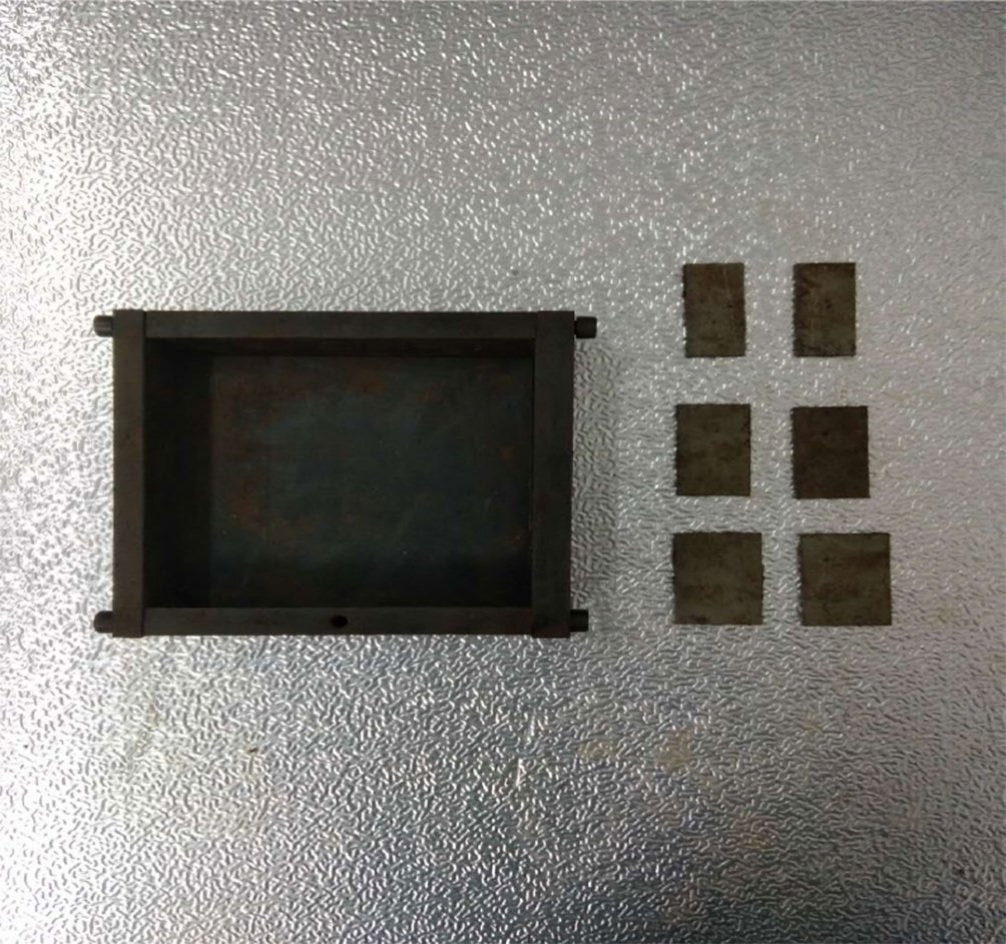
Mould for casting specimen and six steel sheets used to form cracks in specimen.

**Figure 2:**
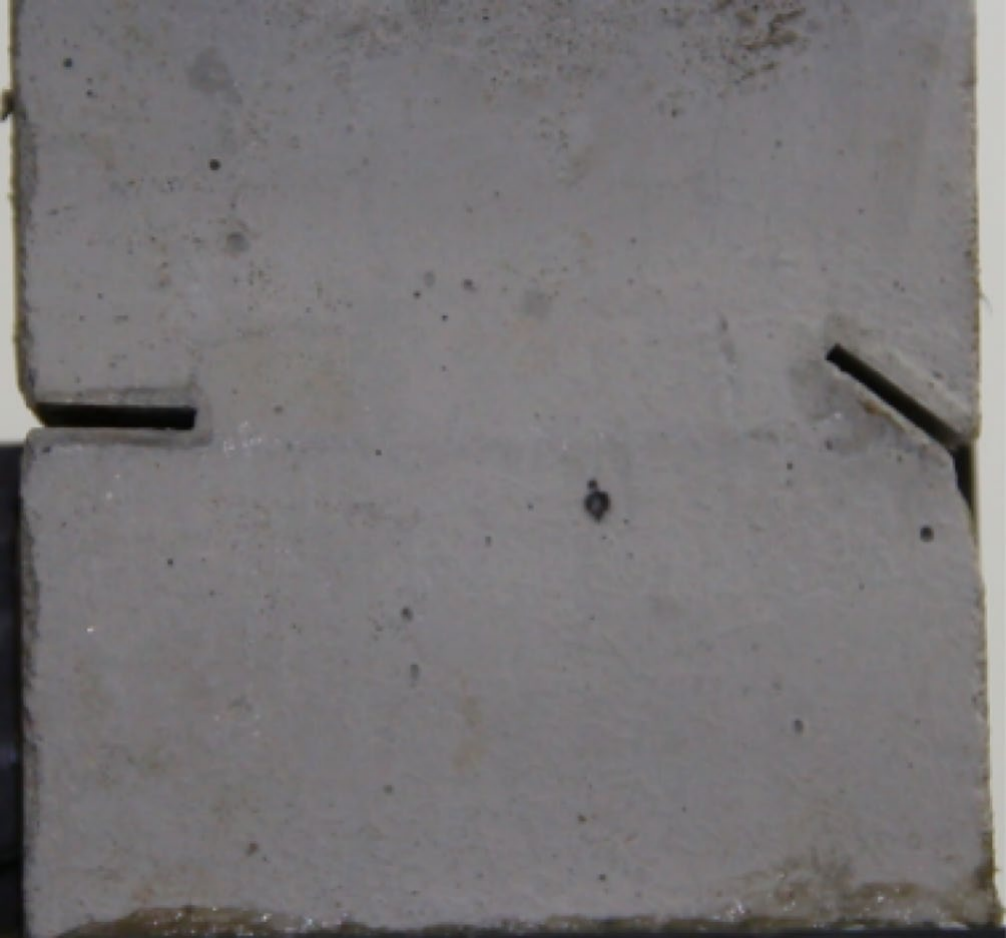
Finished specimen before testing.

The tested specimens were 120 × 120 × 30 mm in size. The tested specimens were comprised of C425 cement, sand, and water in a weight ratio of 1:1:0.7. The specimens also contained a rapid setting agent. The sand was sieved to three particle sizes, 0–0.6 mm, 0.6–1 mm, and 1–2.56 mm. Crack were cast into each specimen and the crack were formed by inserting a steel sheet into the cement–sand–water mix before the specimen solidified in the mould. The steel sheets were 45 mm long; 20, 25, or 30 mm wide; and 2.5 mm thick. The specimen preparation procedure was as follows. First, equal weights of cement and sand were mixed and stirred evenly, then the appropriate amount of water was added. After the components were mixed again, the steel sheets were placed at the proper positions and angles in the mould. After that, the mould was filled with the cement mortar mix layer by layer. Each specimen remained in its mould for 48 h to harden before it was taken out of the mould. The specimens were then cured for 15 days (watered frequently) before testing. The moulds for making the samples were shown in Fig. 1. The test specimen was shown in Fig. 2.

In order to investigate how different fracture patterns affected the fracture of the specimens, seven specimens with different fracture patterns were prepared.

Three specimens with different particle size compositions were prepared in order to investigate the effect of different particle size compositions on the fracture characteristics, acoustic emission properties and mechanical characteristics of the specimens. The first specimen was made of sand in the range of 1–2.56 mm. The second specimen was made from a mixture of sand in the 0–0.6 mm range and the 1–2.56 mm range. The third specimen was made from a mixture of sand in the 0–0.6 mm, 0.6–1.0 mm and 1–2.56 mm ranges. This group of specimens represents rock masses with different non-homogeneities.

### Test method

**Figure 3:**
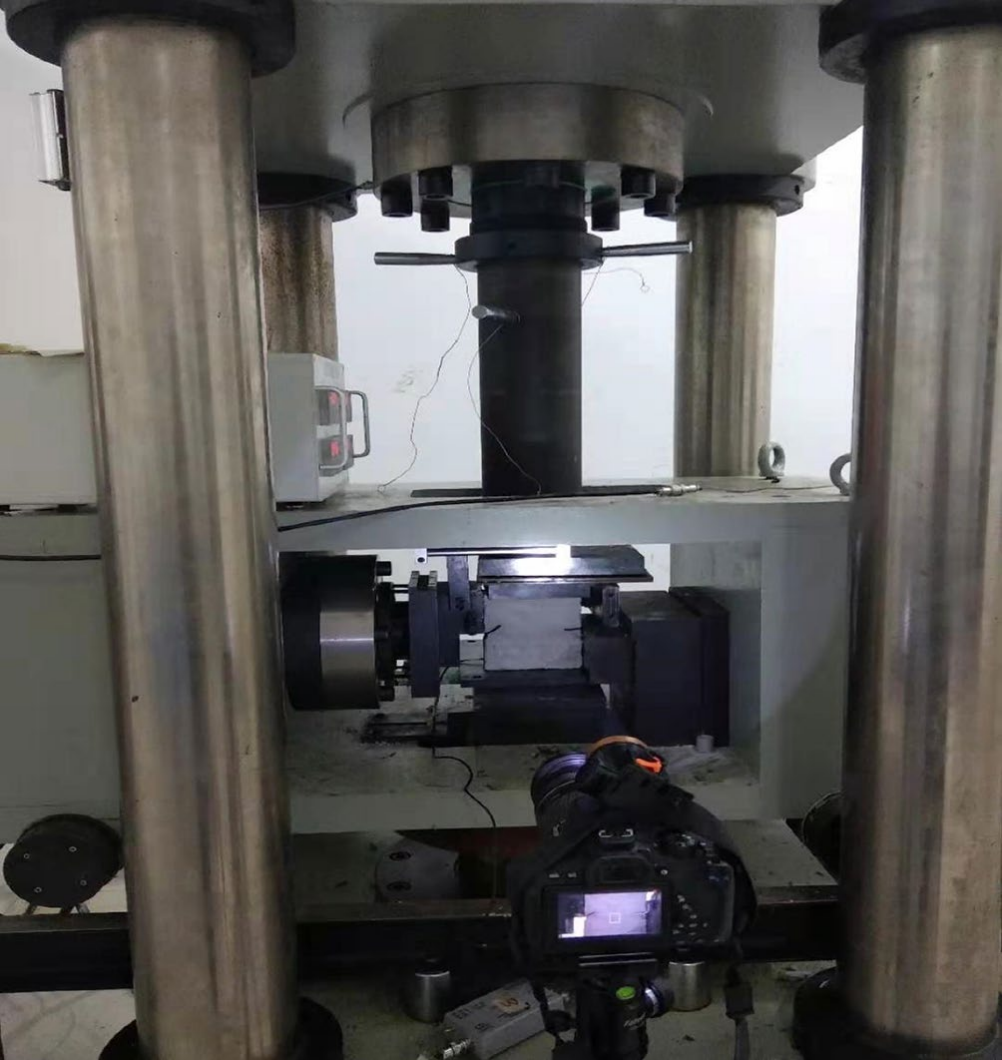
Photograph of the compression shear test showing a specimen ready to be tested.

The top, bottom, left and right surfaces of each specimen were coated with petroleum jelly to reduce end effects. The same horizontal displacement rate of 0.45 mm/min was used for each compression shear test. An acoustic emission monitoring probe was used to record the acoustic emission during fracture of the rock. A high definition camera was used to film the extension of the fracture and the shearing of the rock mass. The lateral displacement and shear of the rock mass during the test were recorded. A servo press, camera and acoustic emission monitor start recording data simultaneously. The experimental equipment is shown in Fig. 3.

## Experimental results and analysis

### Precast crack orientations and their influence on fracture characteristics

#### Precast crack orientations and their influence on fracture pattern

**Figure 4:**
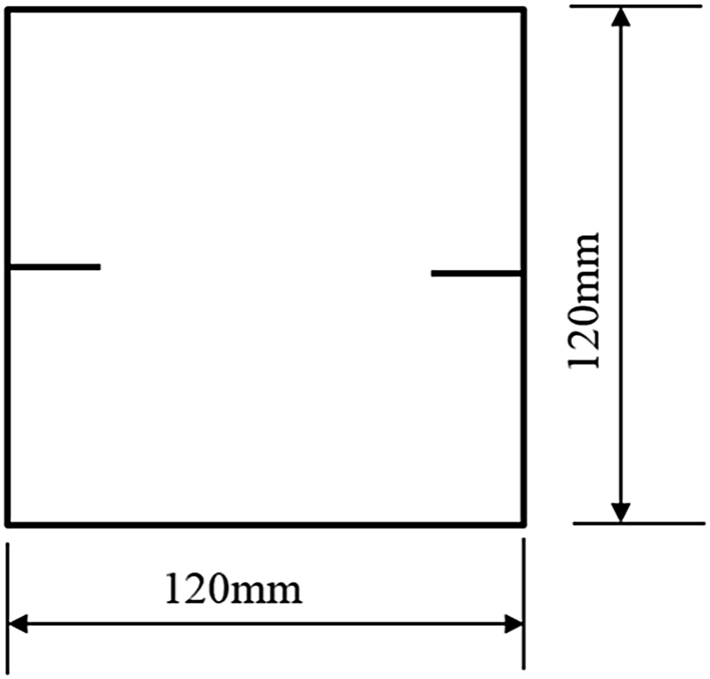
Precast crack orientation 1. Fissure length 2 cm.

**Figure 5:**
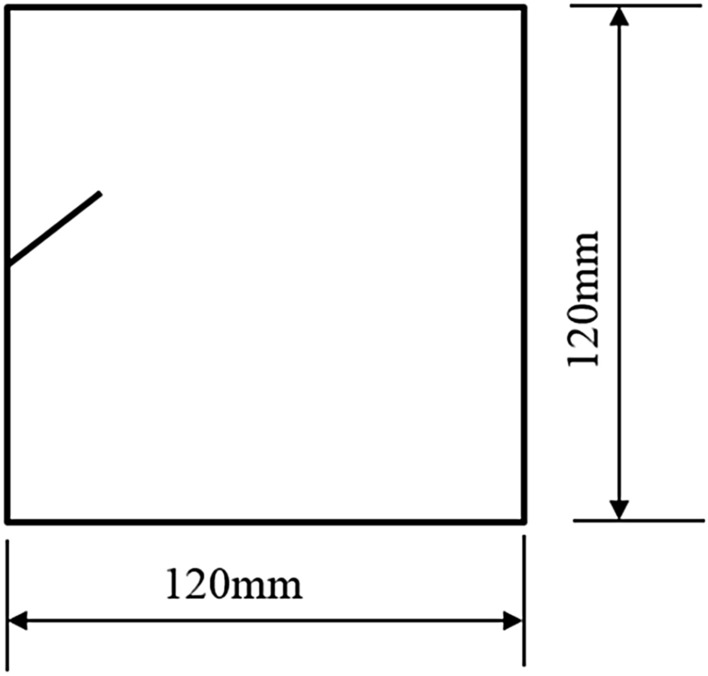
Precast crack orientation 2. Fissure length 2 cm, horizontal angle of fissure 40°.

**Figure 6:**
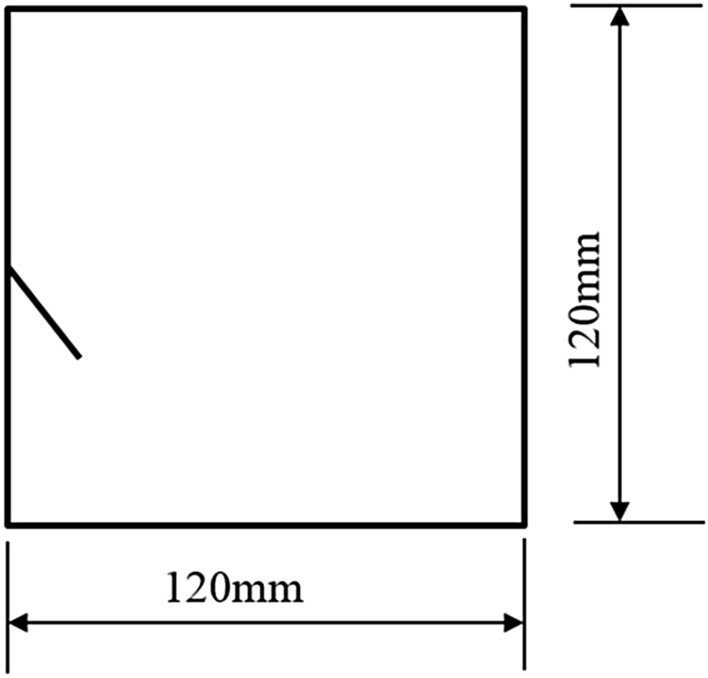
Precast crack orientation 3. Fissure length 2 cm, horizontal angle of fissure 40°.

**Figure 7:**
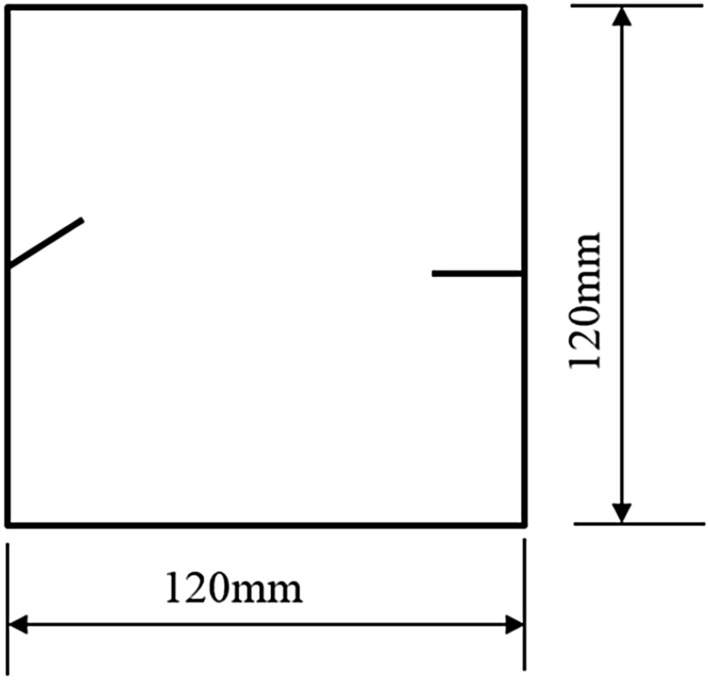
Precast crack orientation 4. Fissure length 2 cm, horizontal angle of fissure 40°.

**Figure 8:**
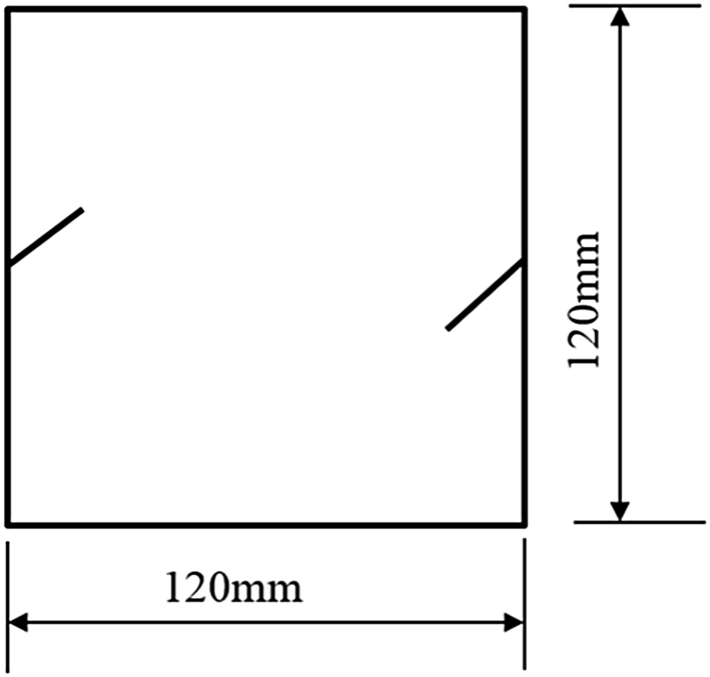
Precast crack orientation 5. Fissure length 2 cm, horizontal angle of fissure 40°.

**Figure 9:**
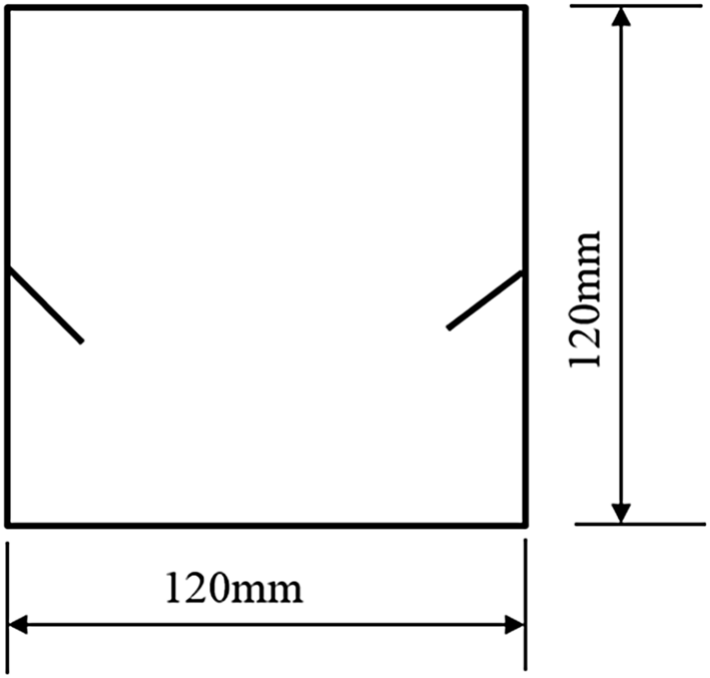
Precast crack orientation 6. Fissure length 2 cm, horizontal angle of fissure 40°.

**Figure 10:**
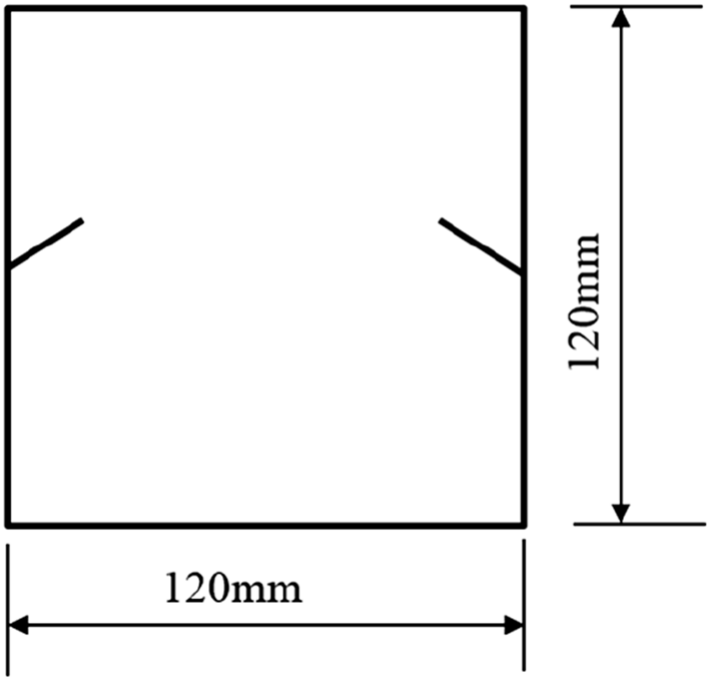
Precast crack orientation 7. Fissure length 2 cm, horizontal angle of fissure 40°.

To study how crack orientations affected rocks fractured under compression shear, seven different precast crack orientations were tested. These orientations are shown in Figs. 4, 5, 6, 7, 8, 9 and 10.

**Figure 11:**
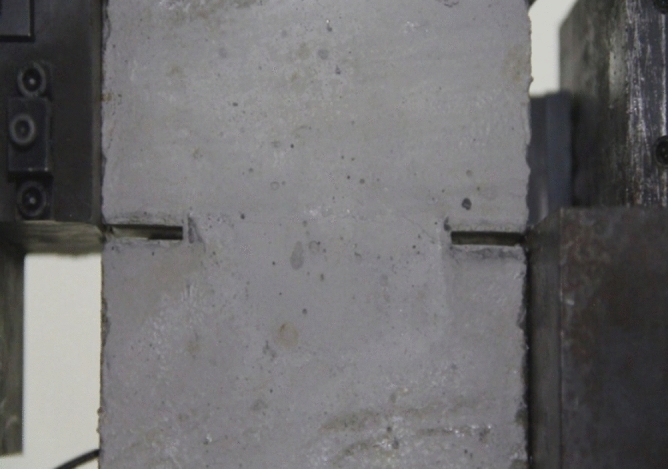
Photograph of a specimen showing fractures appearing.

**Figure 12:**
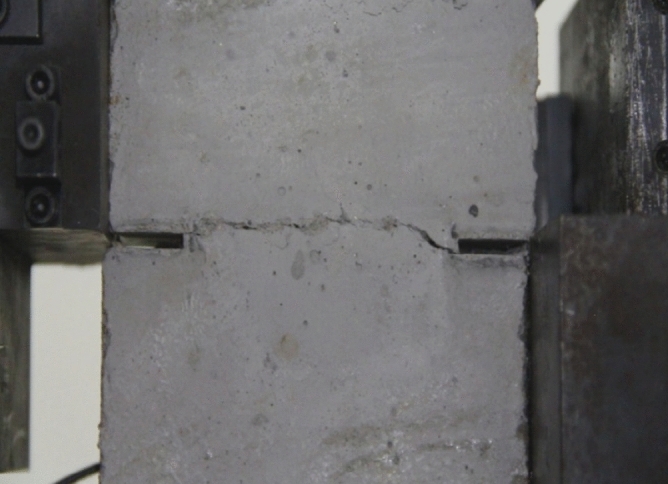
Photograph of a specimen showing fractures penetration.

1. Figures 11 and 12 show photographs of a specimen with orientation 1 cracks being fractured under compression shear.

As can be seen from Figs. 11 and 12, under the action of compression and shear stress, the stress concentration at the crack tip. The crack was produced in the crack tip, and then extended gradually to the other side of the crack. As the shear force increased, the crack gradually became longer and wider. Finally the crack was gradually connected to the other side of the crack tip, forming a through crack. The specimen was completely sheared bad. The crack interface was rough and was a shear crack.

**Figure13:**
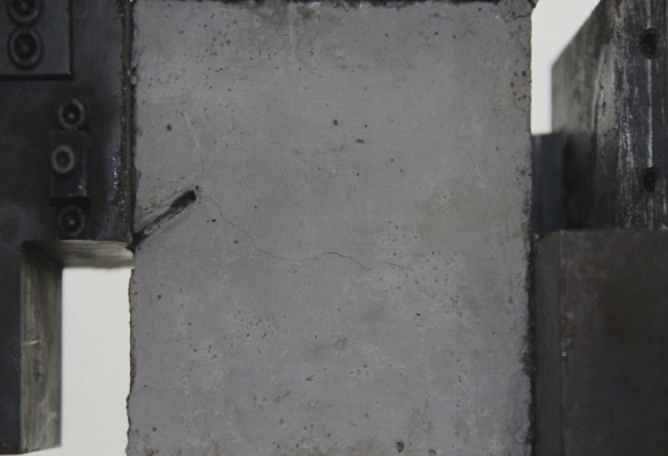
Photograph of a specimen showing fractures appearing.

**Figure 14:**
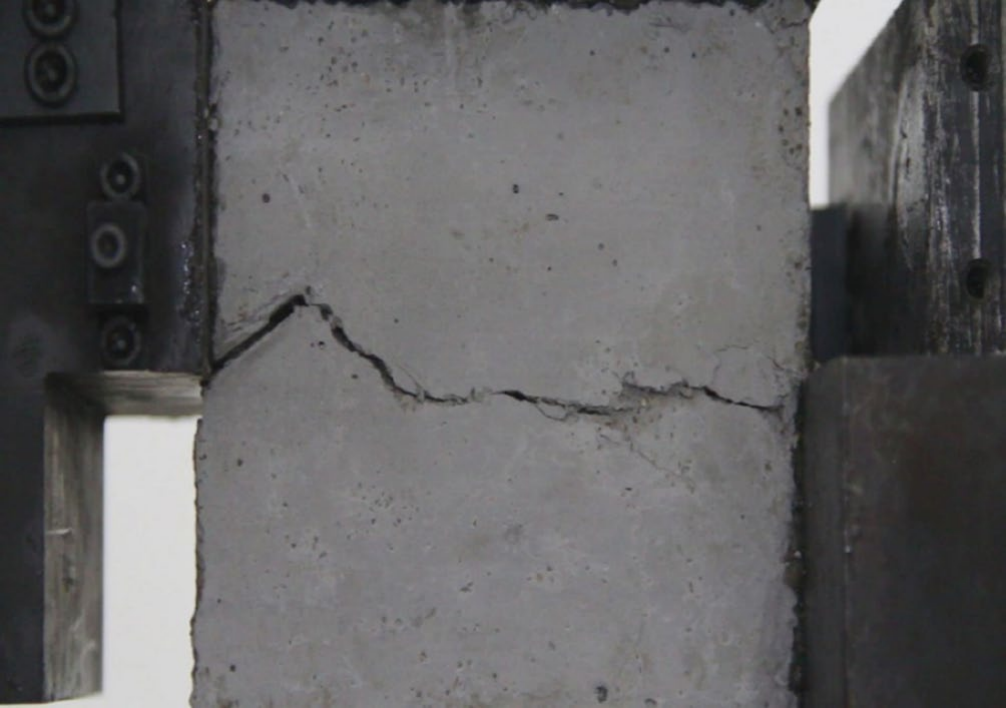
Photograph of a specimen showing fractures penetration.

2. Figures 13 and 14 show photographs of a specimen with orientation 2 cracks being fractured under compression shear.

**Figure15:**
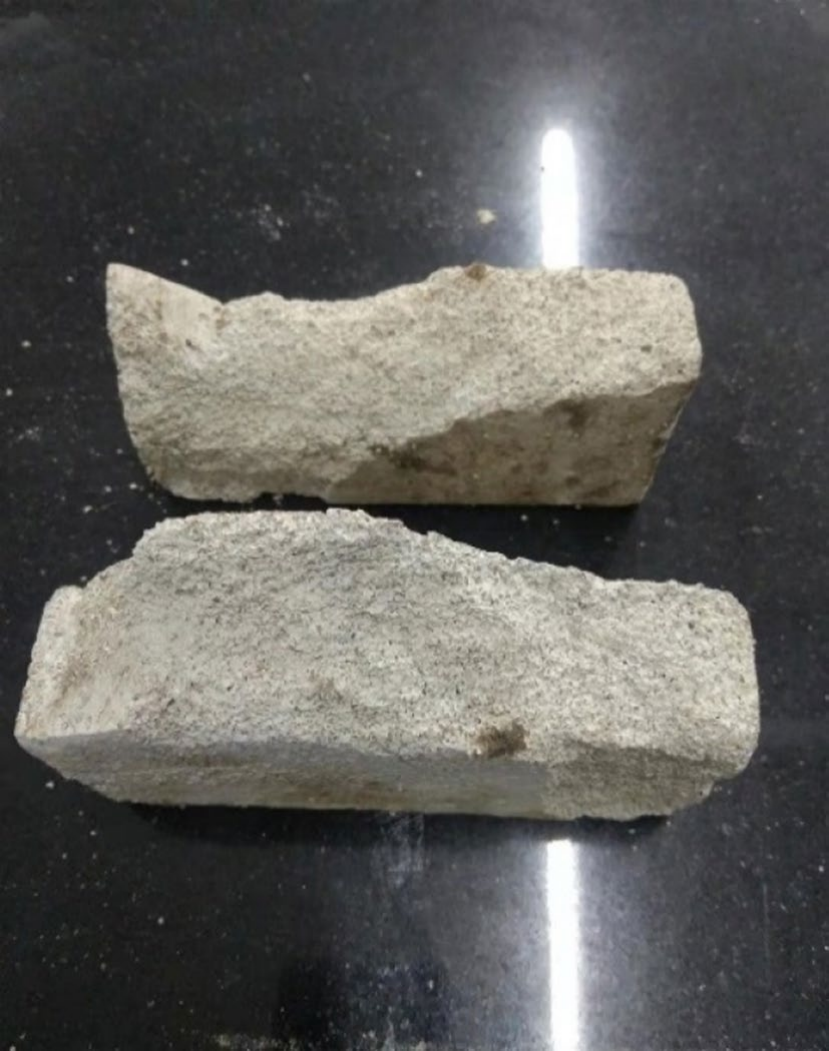
The shear fracture surface of the specimen.

As can be seen from Figs. 13, 14 and 15, under the action of compressive shear stress, the stress was concentrated at the tip of the fracture. The crack was created at the tip of the fissure and then expanded to the lower right. As the shear force increased further, the crack gradually expanded horizontally to the right. The crack gradually became wider and longer. The crack expanded to the right boundary of the rock body to form a through crack, and the specimen was completely sheared. Branch cracks appeared during the expansion of the main crack to the right, and the branch cracks also expanded to the right. The crack interface was rough and was a shear crack.

The shear fracture surface of this specimen showed that the shear fracture surface was rough. The shear fracture face had a prefabricated fracture face at its initiation and was irregularly shaped.

**Figure 16:**
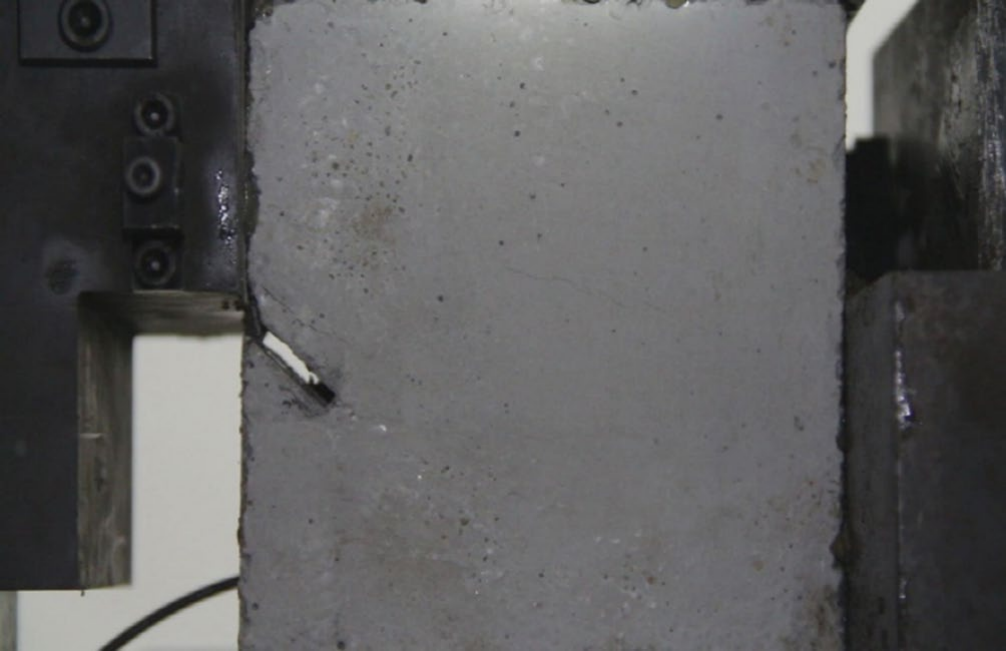
Photograph of a specimen showing fractures appearing from the left boundary of the rock mass.

**Figure 17:**
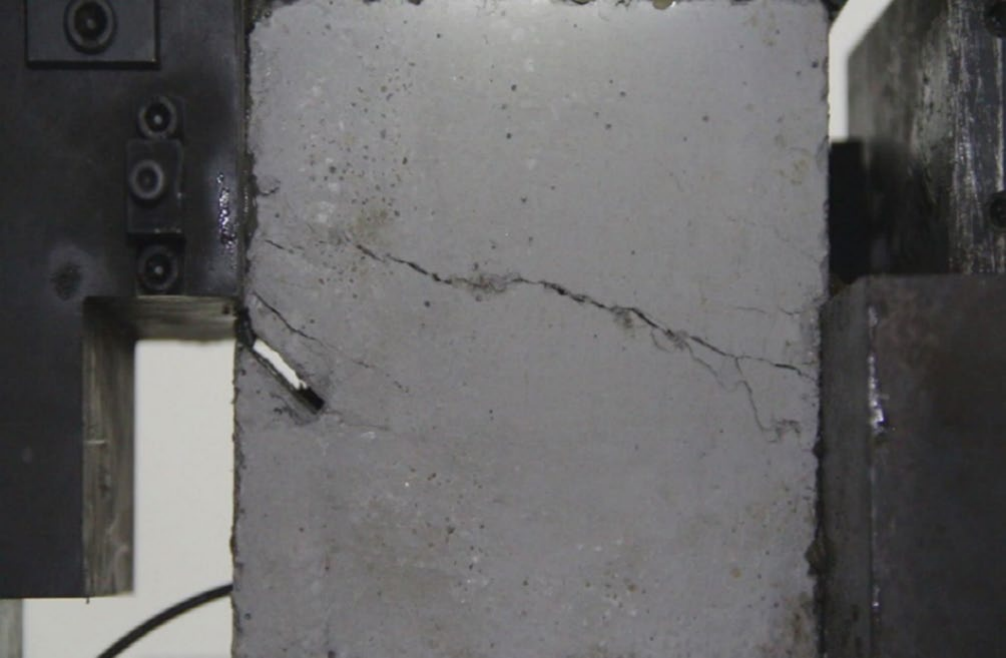
Photograph of a specimen showing fractures penetration.

3. Figures 16 and 17 show photographs of a specimen with orientation 3 cracks being fractured under compression shear.

As can be seen from Figs. 16 and 17, the majority of the left precast fracture was below the horizontal shear. It was less affected by shear and was mainly affected by axial pressure. Instead of cracking from the tip of the precast fissure, multiple cracks were produced at the upper left boundary of the rock mass. As the shear force increased further, the cracks gradually became wider and longer. Eventually it extended to the right boundary of the rock mass to form a through crack and the rock mass sheared badly. The crack interface was rough and was a shear crack.

**Figure 18:**
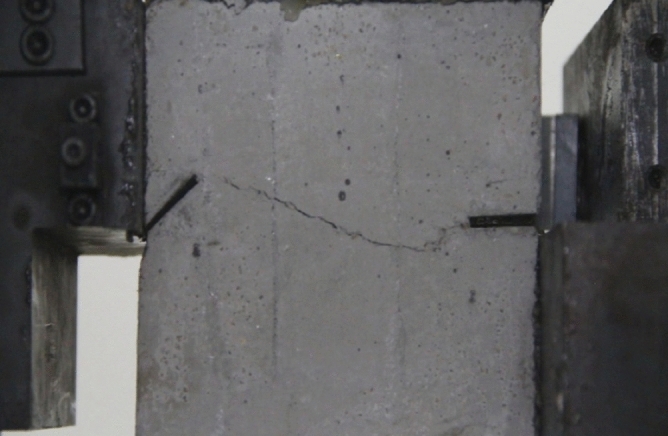
Fracture evolution diagram of fractured rock mass, fractures appeared.

**Figure 19:**
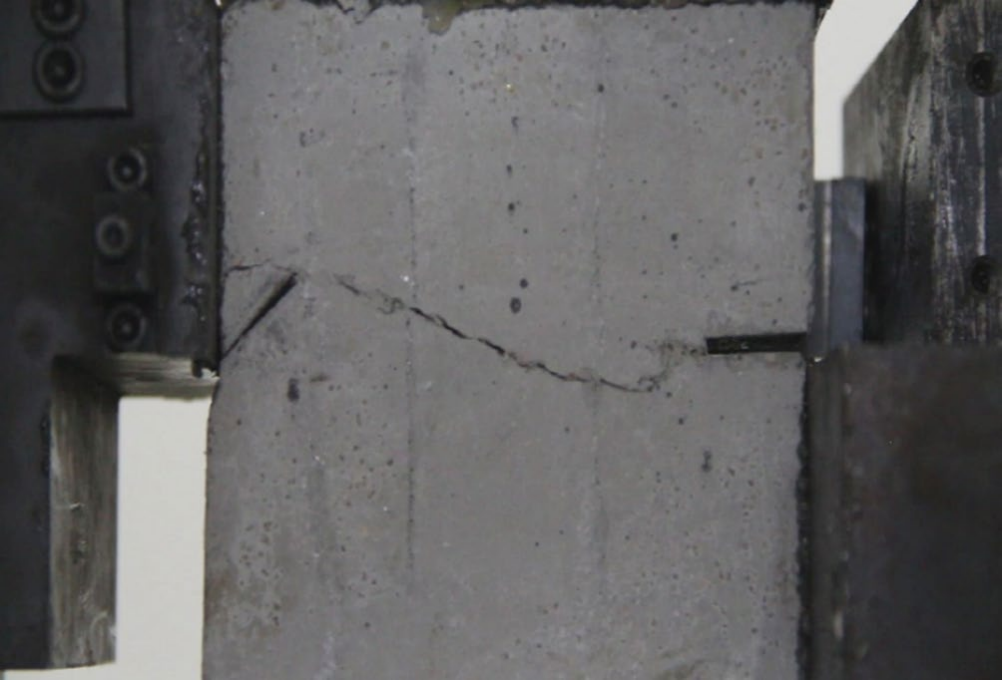
Fracture evolution diagram of fractured rock mass, fractures penetration.

4. Figures 18 and 19 show photographs of a specimen with orientation 4 cracks being fractured under compression shear.

In this crack distribution pattern, the prefabricated crack tip was subjected to stress concentration by axial pressure and shear force. As can be seen from Figs. 18 and 19, the crack initiated at the crack tip, which then expanded to the other side. As the shear force increased further, the crack gradually became wider and longer. The crack then expanded to connect with the tip of the precast crack on the other side, forming a through crack. The left part of the left precast crack was then sheared off in shear due to the lack of support. The crack interface was rough and was a shear crack.

**Figure 20:**
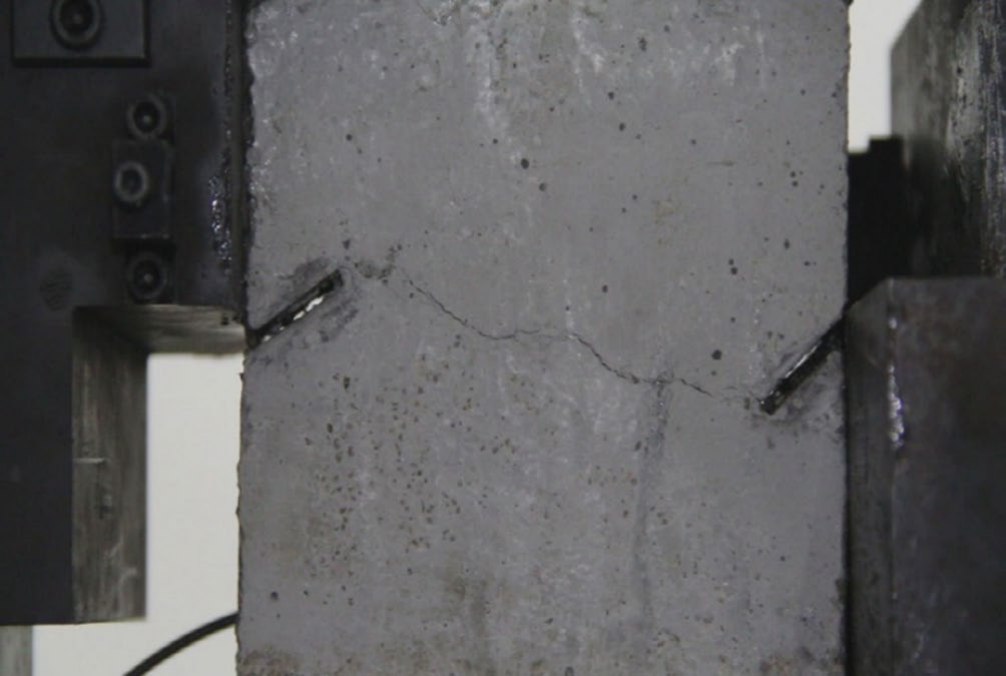
Photograph of a specimen showing fractures appearing.

**Figure 21:**
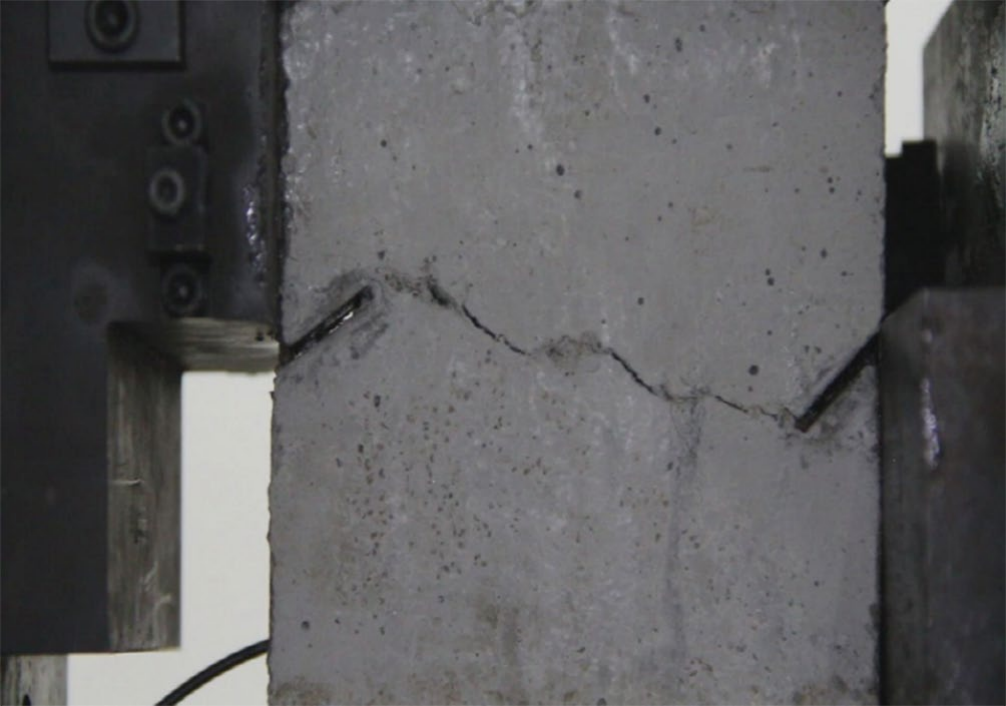
Photograph of a specimen showing fractures penetration.

**Figure 22:**
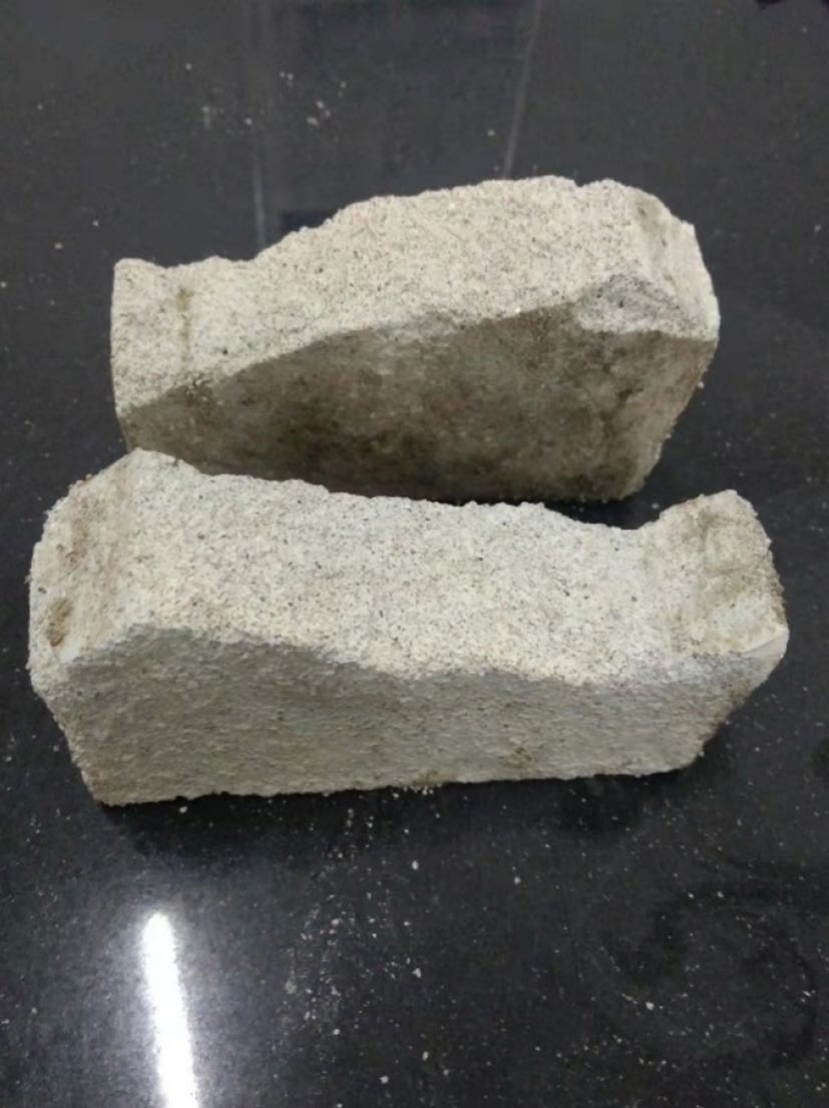
The shear fracture surface of the specimen.

5. Figures 20, 21 and 22 show photographs of a specimen with orientation 5 cracks being fractured under compression shear.

In this fracture mode, the precast fracture tip was subjected to stress concentration by axial pressure and shear. As can be seen from Figs. 19 and 20, the crack started at the tip of the precast fracture and after starting the crack expanded to the other side. As the shear force increased, the crack connected with the precast fracture tip on the other side to form a through crack and the rock sheared badly. The crack interface was rough and was a shear crack.

The shear fracture surface of this specimen showed that the shear fracture surface was rough. The shear fracture face had a prefabricated fracture face at its initiation and was irregularly shaped.

**Figure 23:**
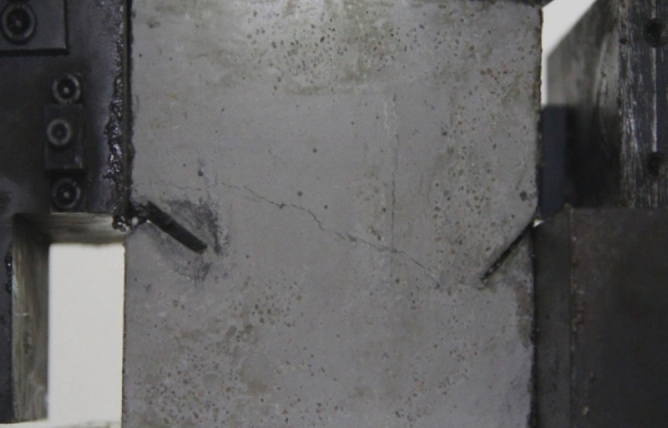
Photograph of a specimen showing fractures appearing in the middle of the rock mass.

**Figure 24:**
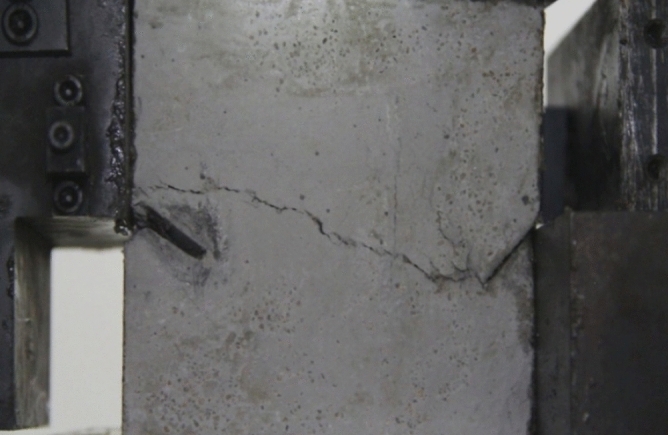
Photograph of a specimen showing fractures propagation.

**Figure 25:**
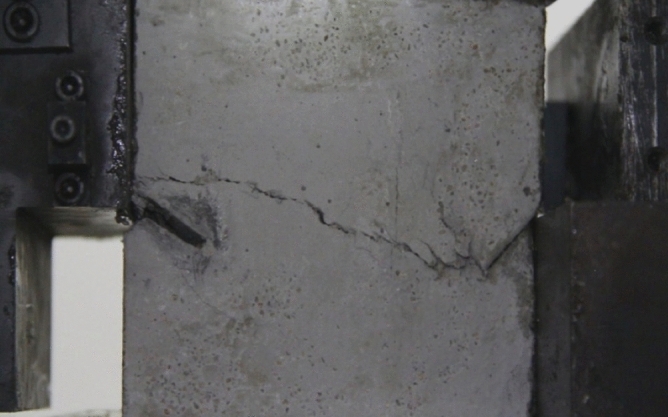
Photograph of a specimen showing fractures propagation.

**Figure 26:**
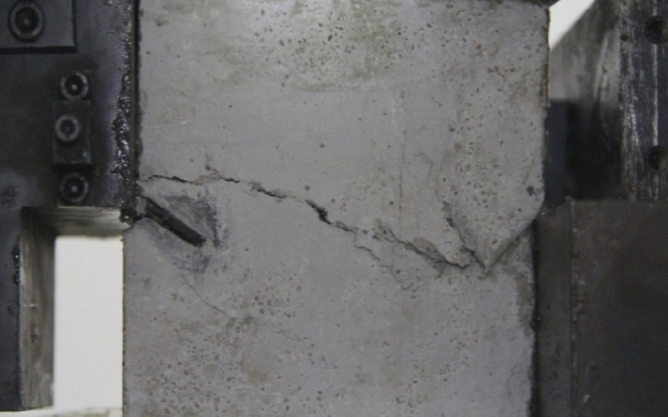
Photograph of a specimen showing fractures penetration.

6. Figures 23, 24, 25 and 26 show photographs of a specimen with orientation 6 cracks being fractured under compression shear.

As can be seen from Figs. 23, 24, 25 and 26, the left precast fracture tip was below the horizontal shear, which was less affected by shear and more affected by axial forces. The rock was subjected to axial pressure and shear and the fracture was created in the middle. As the shear force increased, it gradually extended to the tip of the right fissure and the upper left boundary of the rock mass to form a through fissure and the specimen was completely sheared. The left prefabricated fracture tip was less affected by shear and no cracks were produced at the fracture tip. The precast fracture tip on the right was stressed under pressure and shear, producing a diagonal upward crack. The crack interface was rough and was a shear crack.

**Figure 27:**
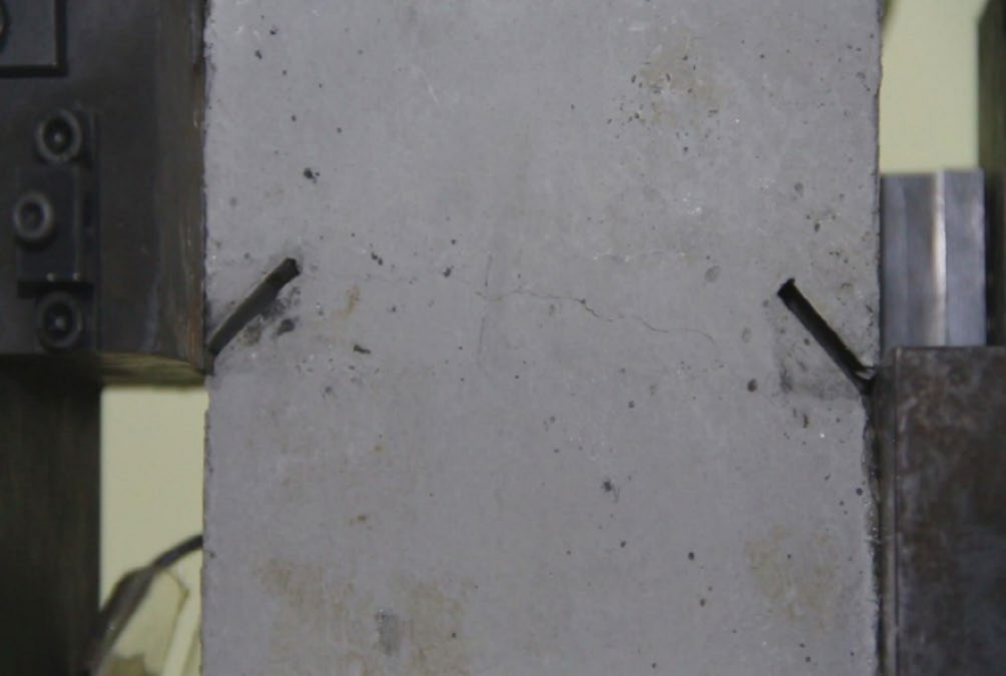
Photograph of a specimen showing fractures appearing in the middle of the rock mass.

**Figure 28:**
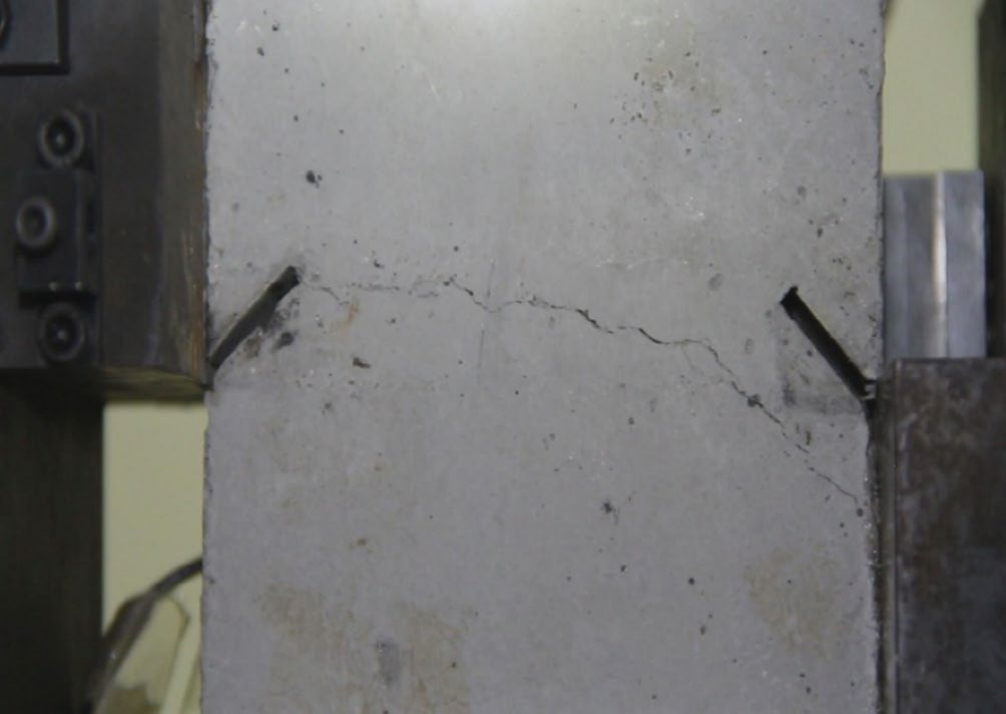
Photograph of a specimen showing fractures propagate in the middle of the rock mass.

**Figure 29:**
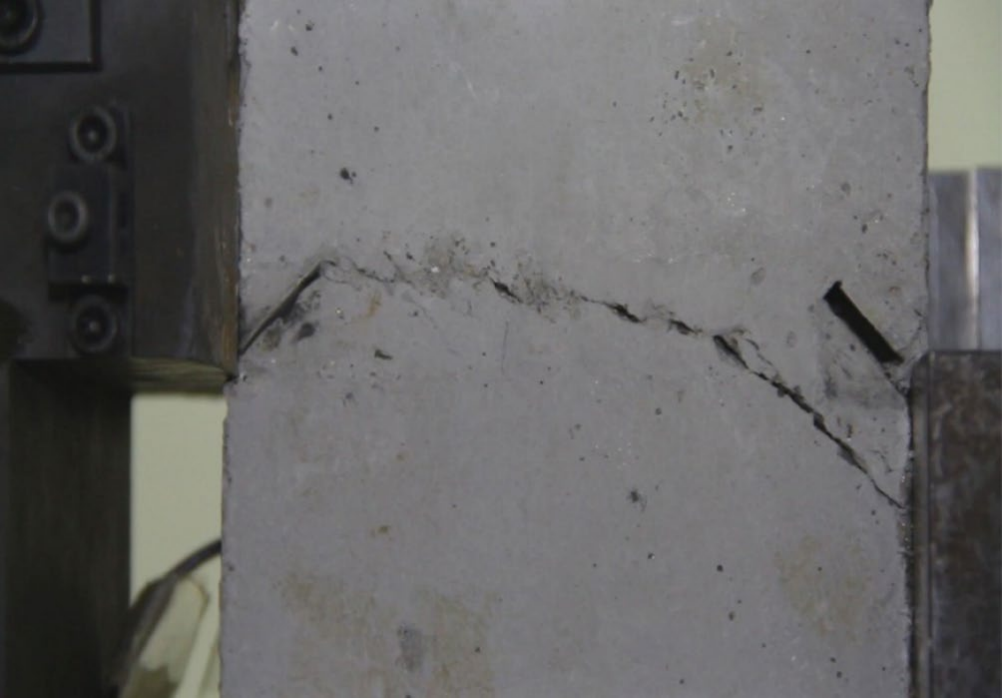
Photograph of a specimen showing fractures propagate in the middle of the rock mass.

**Figure 30:**
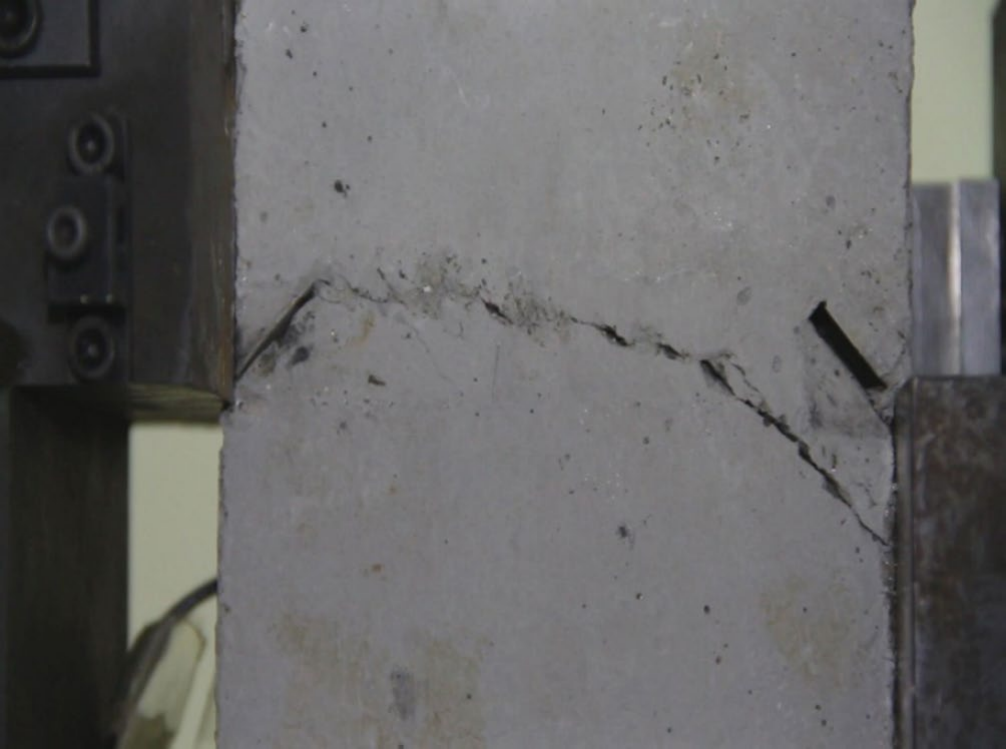
Photograph of a specimen showing fractures penetration.

7. Figures 27, 28, 29 and 30 show photographs of a specimen with orientation 7 cracks being fractured under compression shear.

As can be seen from Figs. 27, 28, 29 and 30, the right-hand prefabricated fracture was mainly subject to axial compression and was less affected by shear forces. No cracks started from the fracture tip. The fractures rose in the middle of the rock mass. The left prefabricated fissure was subject to pressure and shear, with stress concentration at the fissure tip. After generation it expanded to the lower right and upper left, extending to the fissure tip and the lower right boundary of the rock mass, forming through cracks and shear damage to the rock mass. The fracture interface was rough and was a shear fracture.

#### Precast crack orientations and their influence on mechanical properties

**Figure 31:**
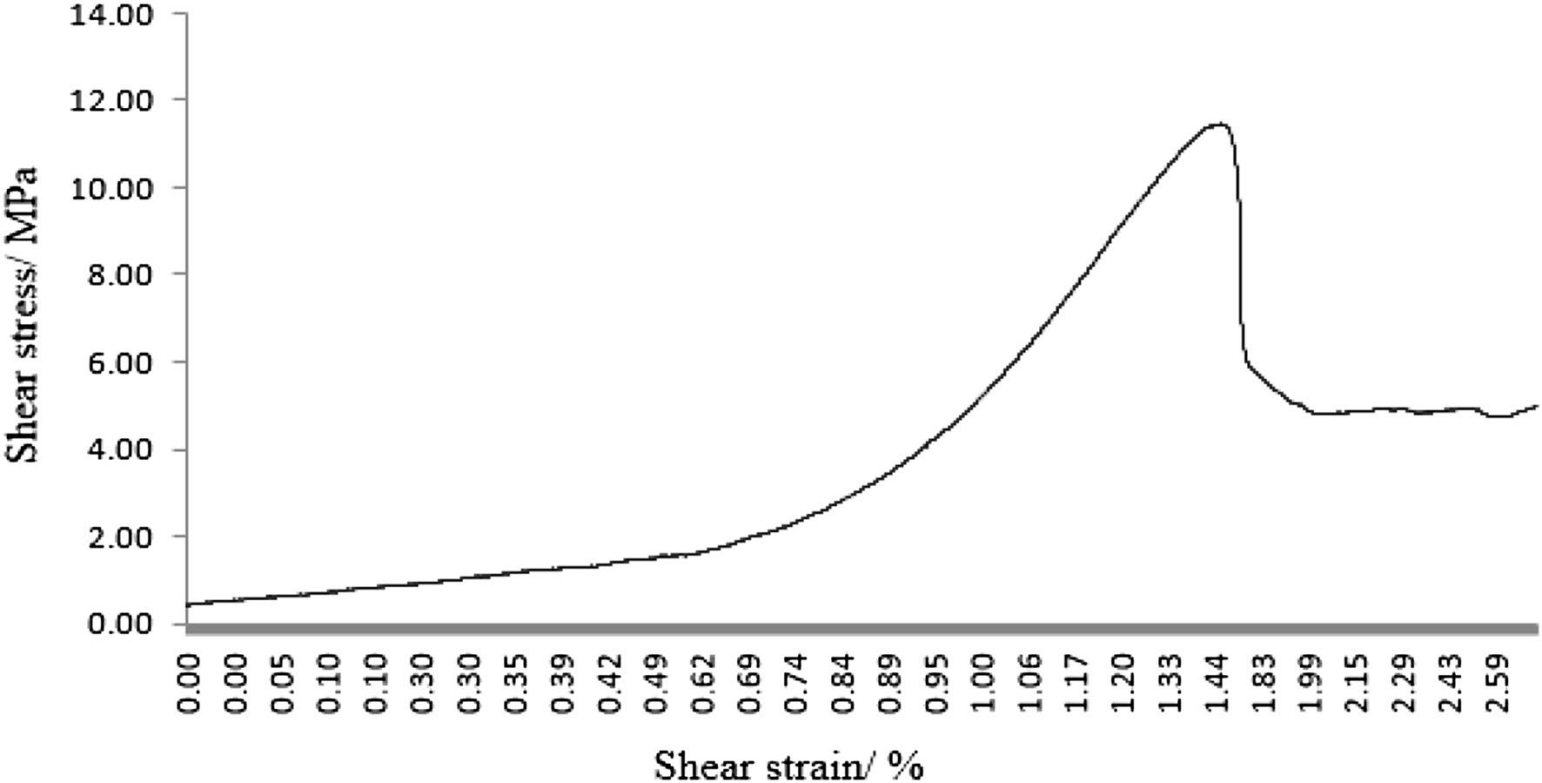
The first fracture mode rock shear stress-shear strain curve.

**Figure 32:**
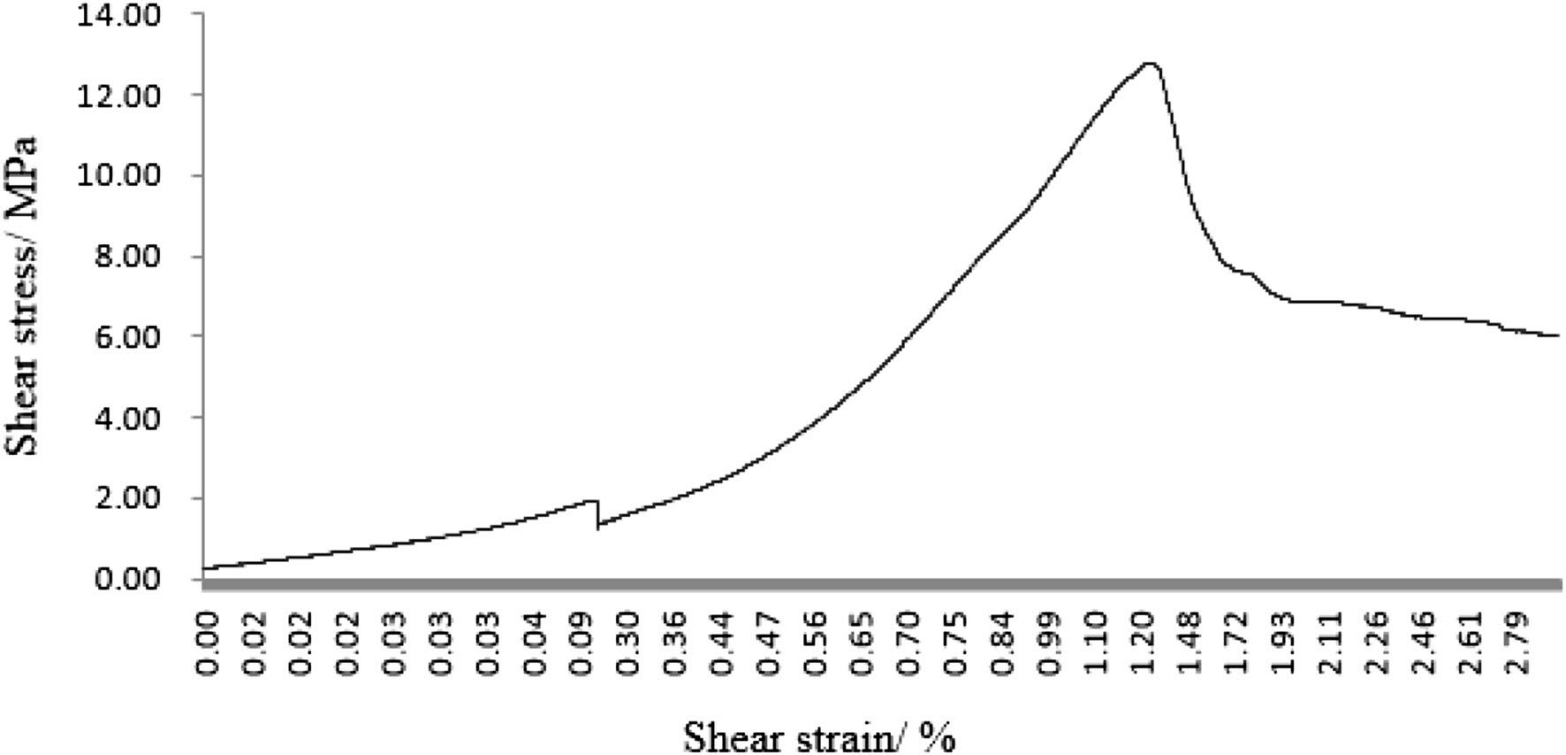
The second fracture mode rock shear stress-shear strain curve.

**Figure 33:**
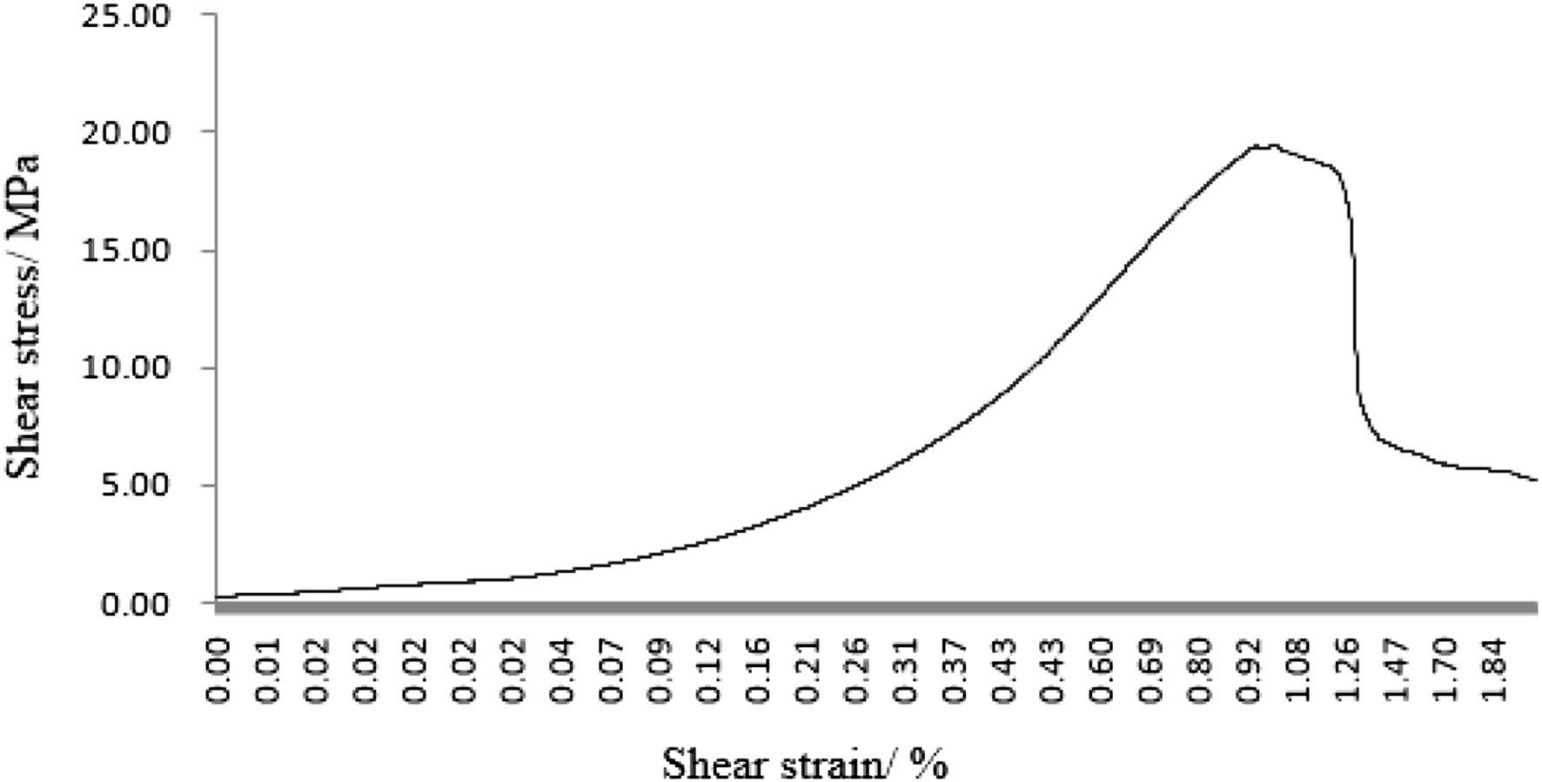
The third fracture mode rock shear stress-shear strain curve.

**Figure 34:**
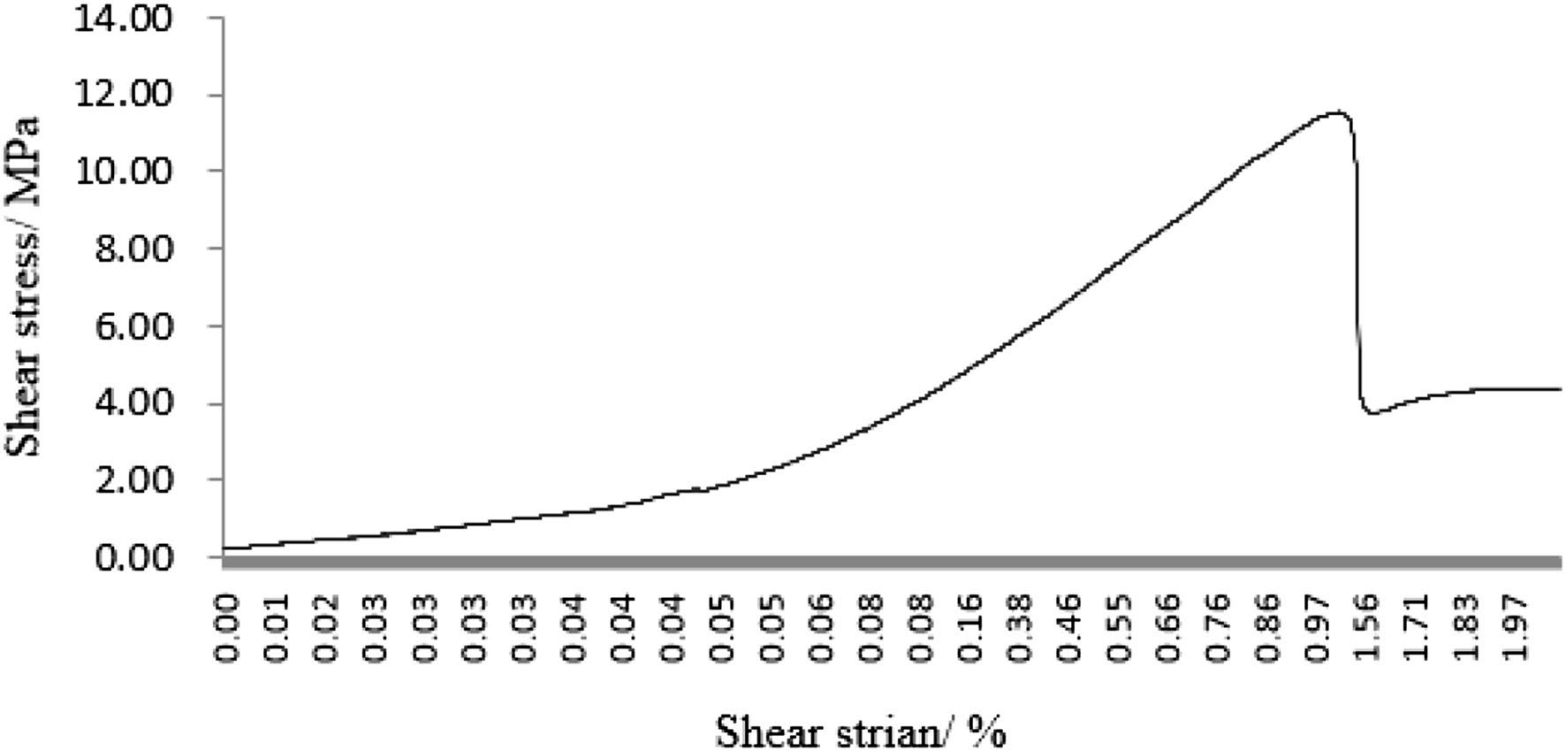
The fourth fracture mode rock shear stress-shear strain curve.

**Figure 35:**
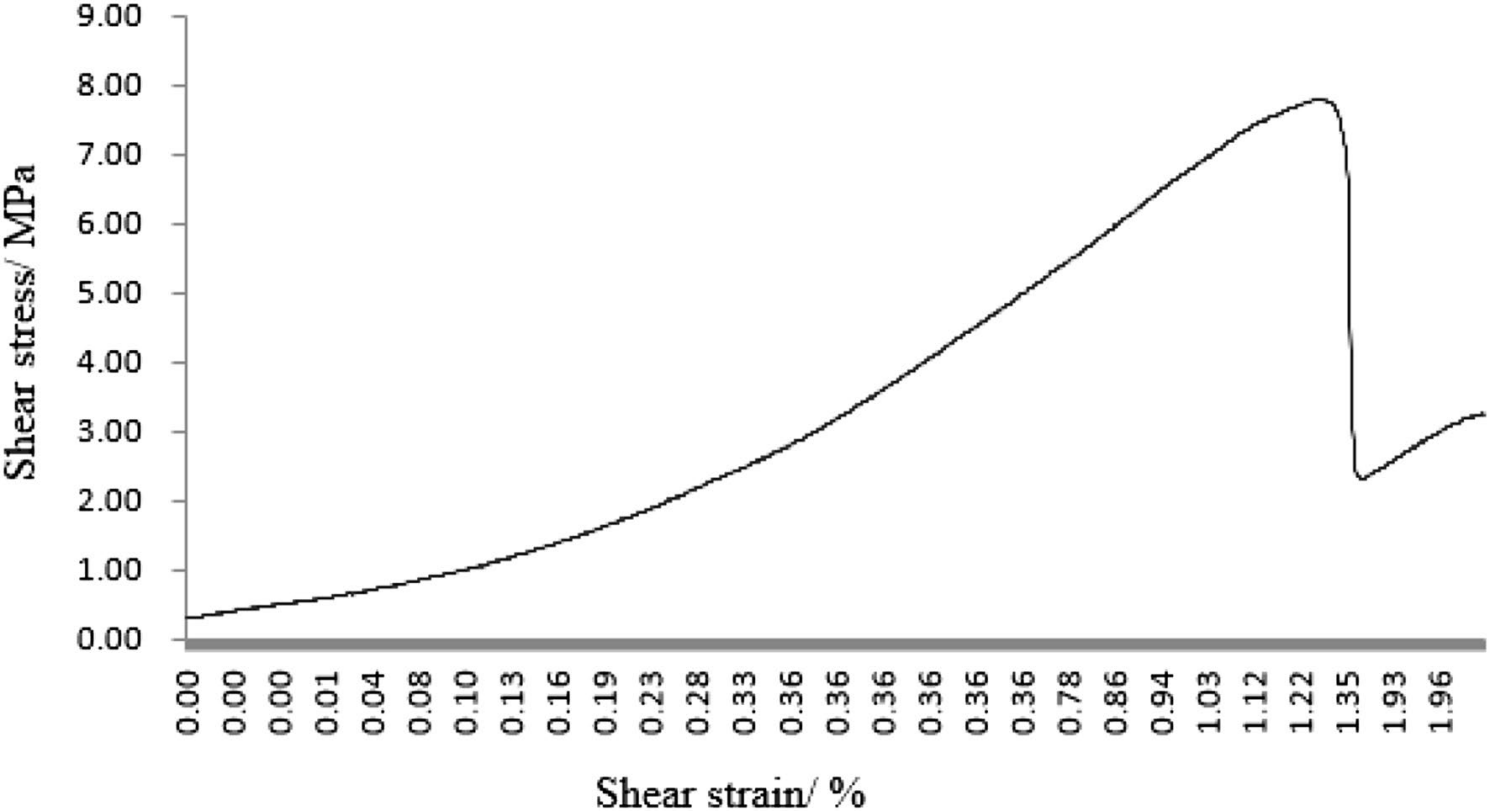
The fifth fracture mode rock shear stress-shear strain curve.

**Figure 36:**
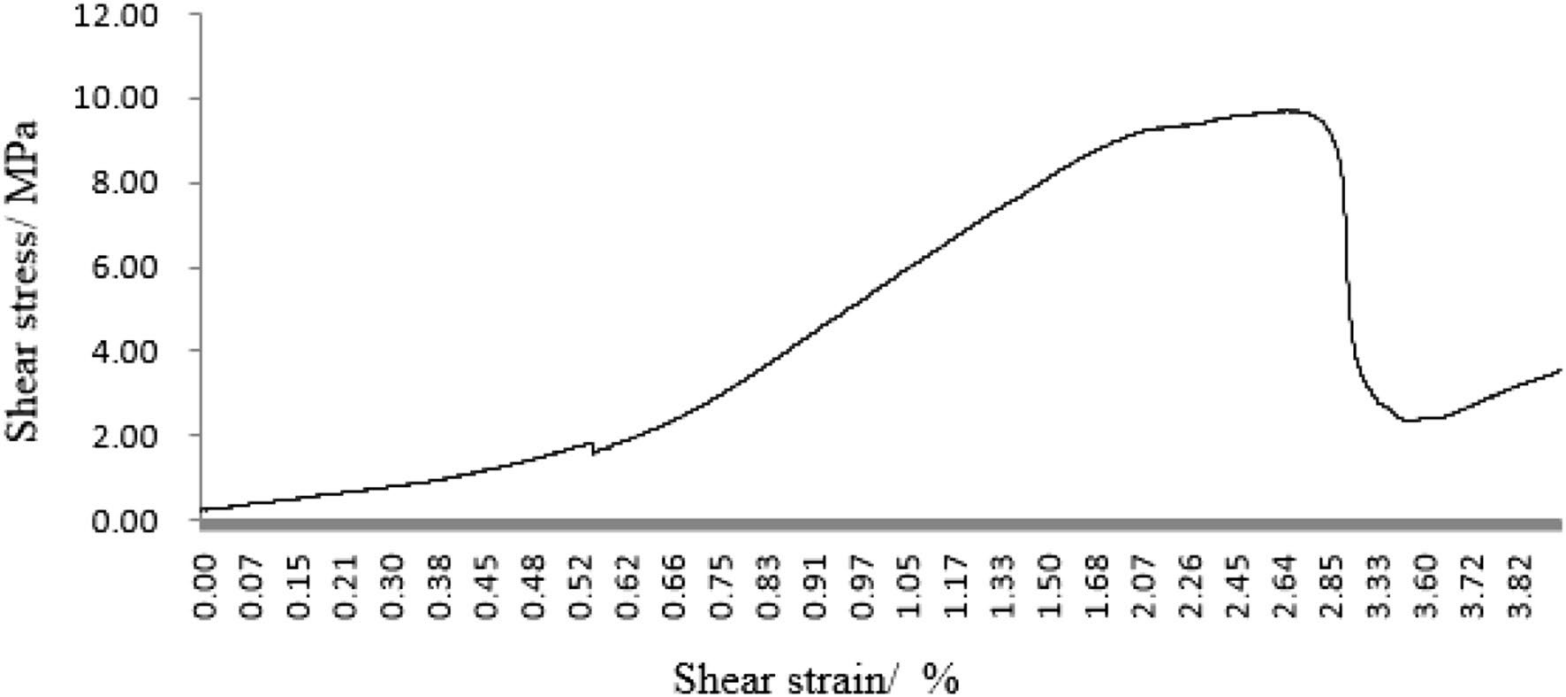
The sixth fracture mode rock shear stress-shear strain curve.

**Figure 37:**
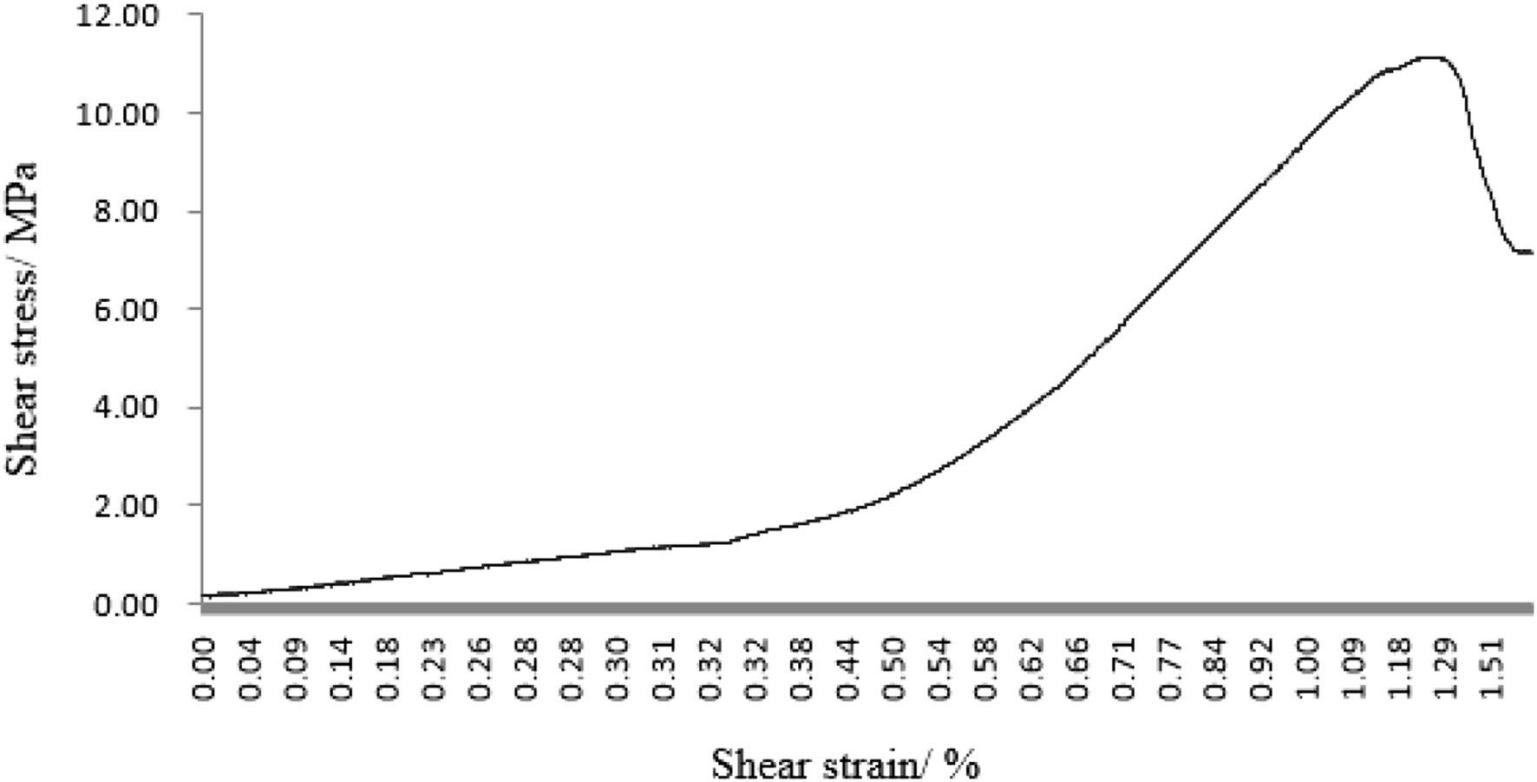
The seventh fracture mode rock shear stress-shear strain curve.

As can be seen from Figs. 31, 32, 33, 34, 35, 36 and 37, the first specimen, the fourth specimen and the fifth specimen all showed a straight drop in shear after reaching peak shear. The specimens underwent brittle fracture with rapid formation of large macroscopic cracks.

The strength of the specimens rapidly decreased. The second specimen, the third specimen, the sixth specimen and the seventh specimen did not show a linear decrease in shear stress after reaching the peak shear stress, but a gradual decrease with increasing shear strain. This indicated that the specimens were fractured gradually. The specimens gradually lost strength and the shear stress decreased as fracture proceeded.

After the fourth, fifth and sixth specimens had reached their minimum shear stresses, the shear stress rose again as the shear strain increased. This indicated that although large fractures have formed in these specimens, there were still some minor fractures that have not formed. There was still a certain amount of shear strength preventing further fracture of the specimens. After the shear stresses in the first, second, third, fourth and seventh specimens had reached their minimum values. The shear stress did not rise any further. This indicated that these specimens have been completely sheared.

These specimens have completely lost their strength.

It was clear from the tests that the fracture pattern has a significant influence on the fracture process and mechanical properties of the rock mass.

### Grain size composition on rock fracture characteristics

**Figure 38:**
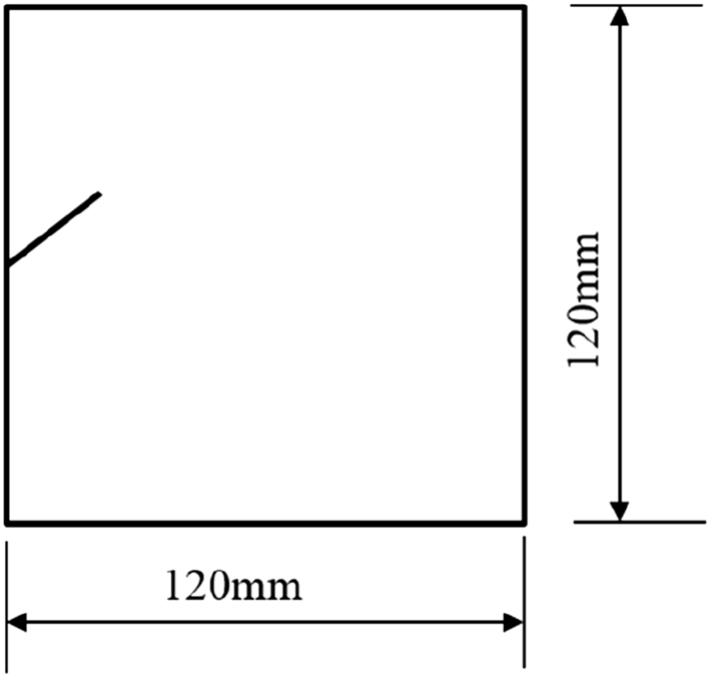
Fissure length 2 cm, horizontal angle of fissure 40°, in specimens with different grain size ranges.

In order to investigate the effect of grain size on the fracture characteristics and mechanical properties of the specimens, specimens with the same fracture pattern were made. However, the specimens were made from sand with different particle size ranges. The first specimen contained sand in the range of 1–2.56 mm. The second specimen contained equal amounts of sand in the 0–0.6 mm range and in the 1–2.56 mm range. The third specimen contained sand from all three ranges of grain sizes. The pattern of prefabricated cracks for these three specimens is shown in Fig. 38.

#### Grain size composition on fracturing

1. Fracturing in a specimen produced from a cement mortar mix containing sand in the 1–2.56 mm grain size range.
2. Fracturing in a specimen produced from a cement mortar mix containing sand in the 0–0.6 mm and 1–2.56 mm grain size range.
3. Fracturing in a specimen produced from a cement mortar mix containing sand in the 0–2.56 mm grain size range.

**Figure 39:**
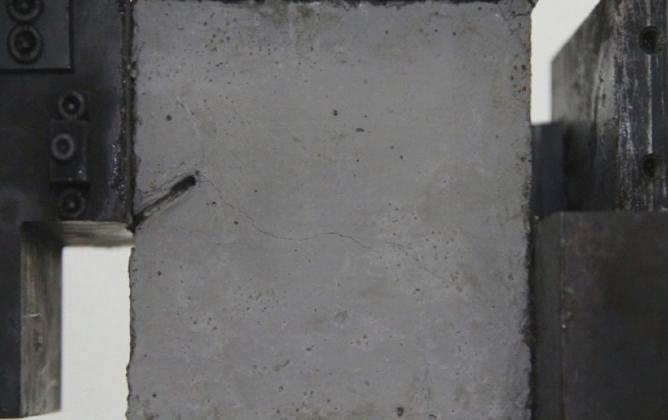
Photograph of a specimen showing fractures appearing.

**Figure 40:**
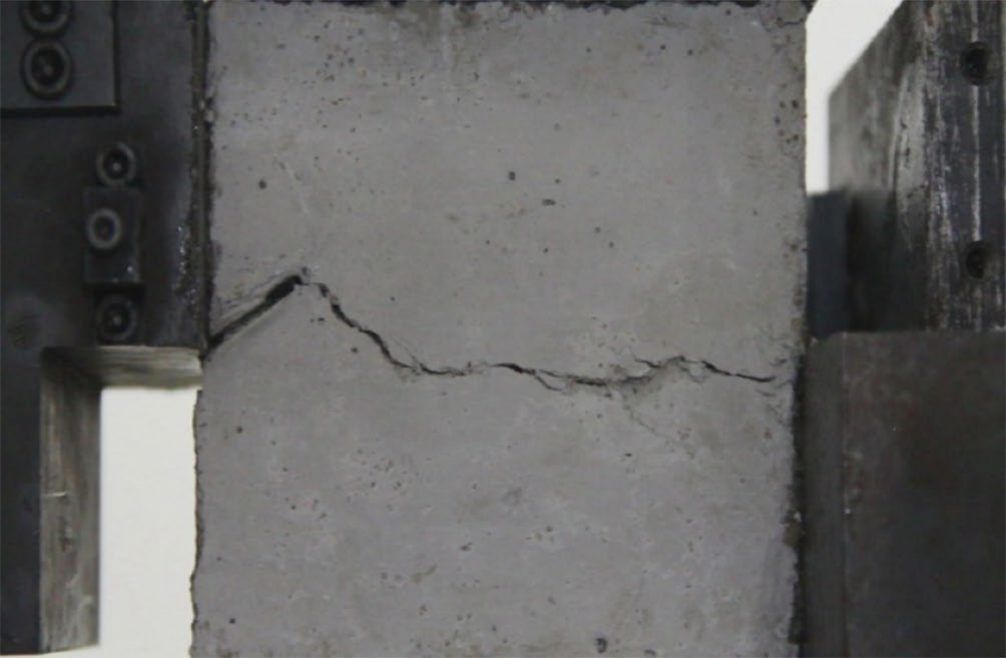
Photograph of a specimen showing fractures penetration.

**Figure 41:**
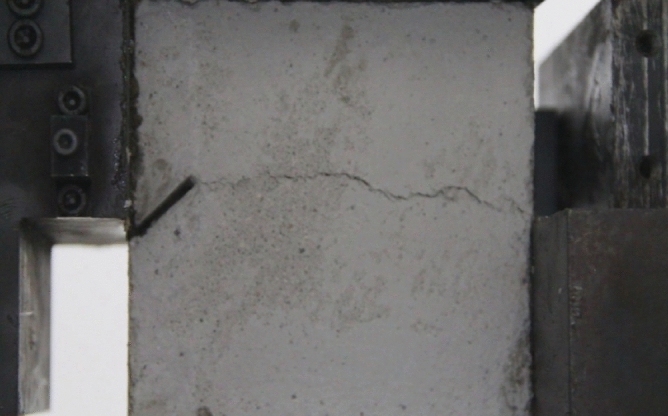
Photograph of a specimen showing fractures appearing.

**Figure 42:**
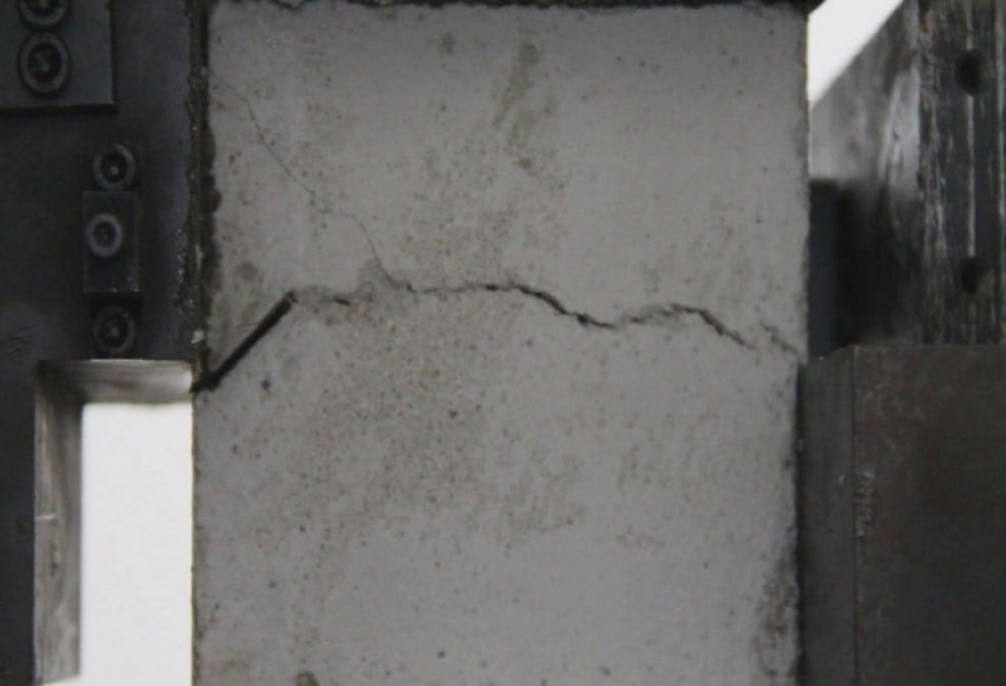
Photograph of a specimen showing fractures penetration.

**Figure 43:**
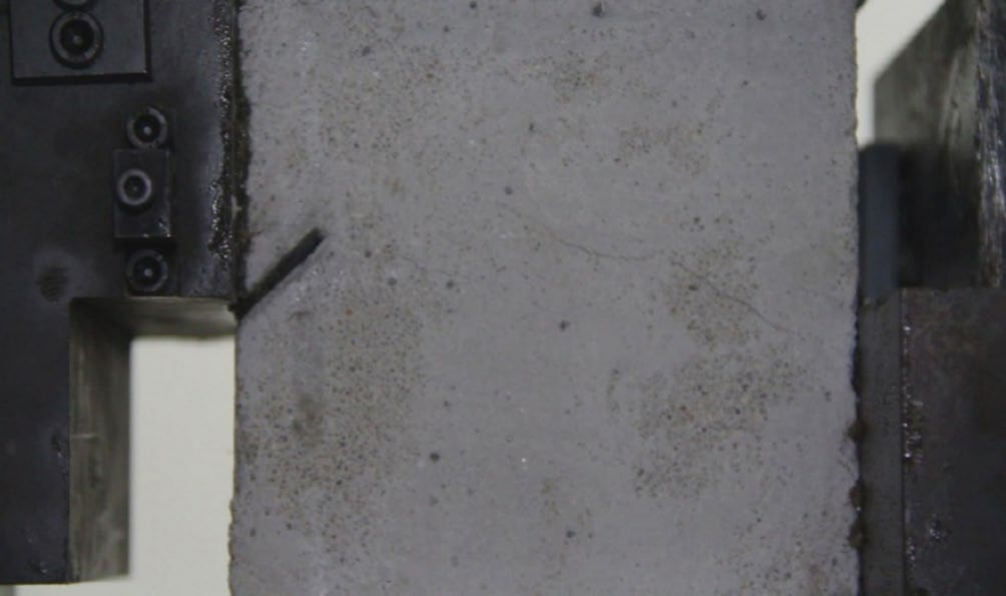
Photograph of a specimen showing fractures appearing in the middle of the specimen.

**Figure 44:**
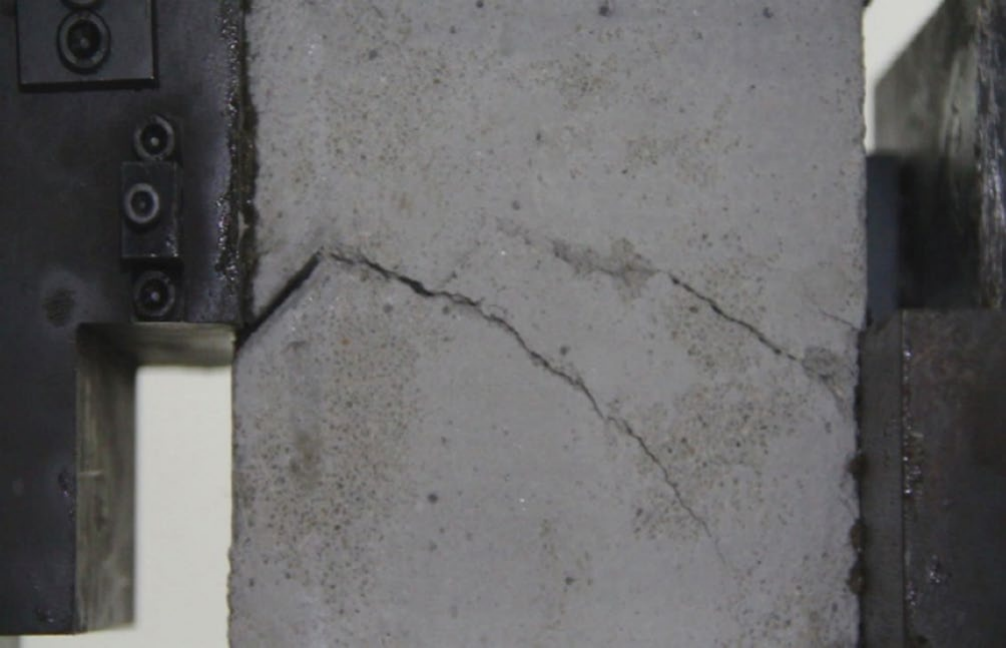
Photograph of a specimen showing fractures propagating and penetration.

As can be seen from Figs. 39, 40, 41, 42, 43 and 44, when comparing the fracture characteristics of specimens composed of different particle size ranges, it can be seen that the location of crack initiation and fracture morphology were different for specimens composed of different particle size ranges.

#### Grain size composition on mechanical properties

**Figure 45:**
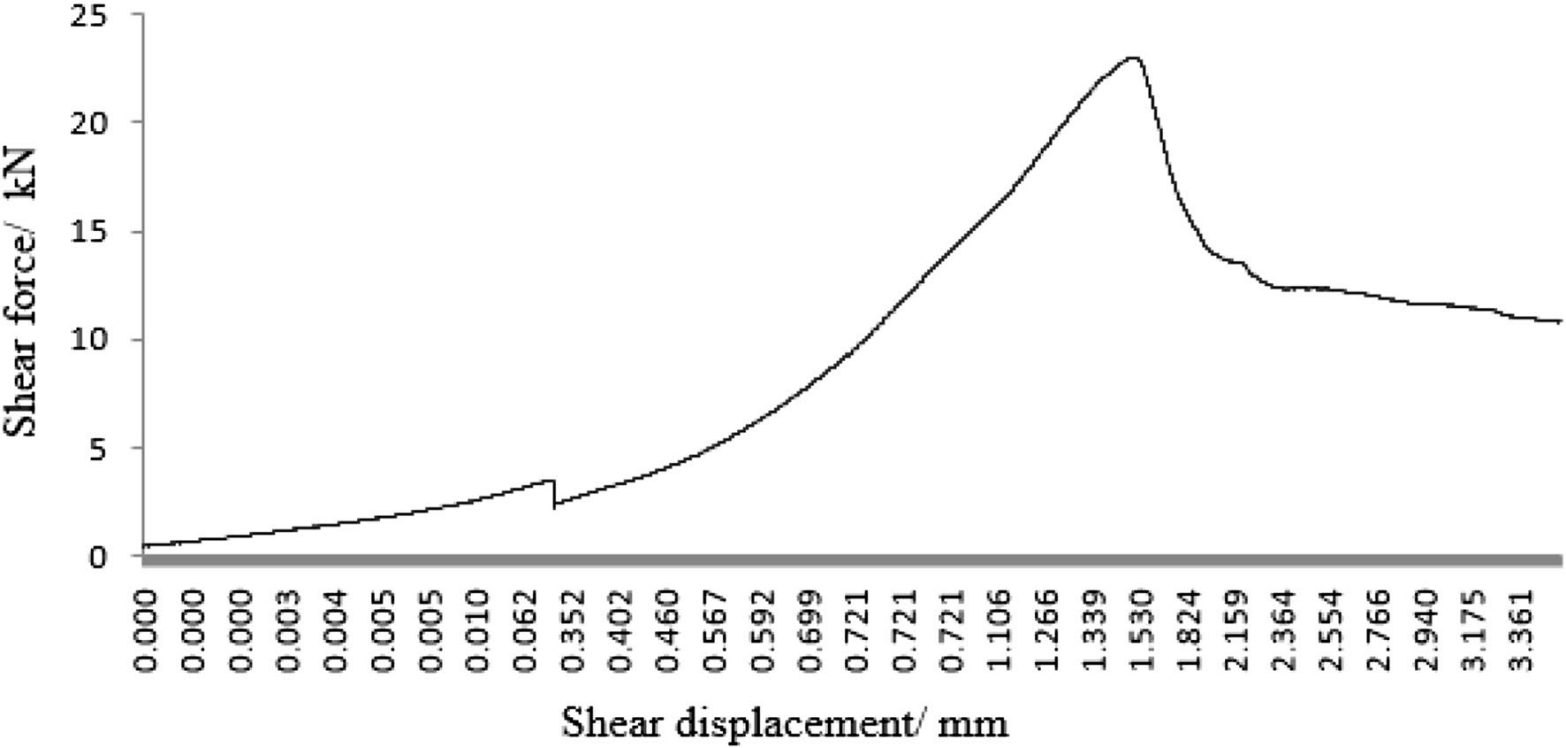
Shear force–shear displacement curve for a specimen containing 1–2.56 mm sand particles (one grain size range).

**Figure 46:**
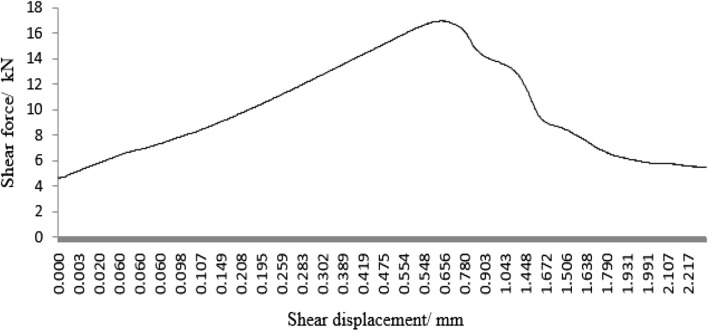
Shear force–shear displacement curve for a specimen containing 0–0.6 mm and 1–2.56 mm sand particles (two grain size ranges).

**Figure 47:**
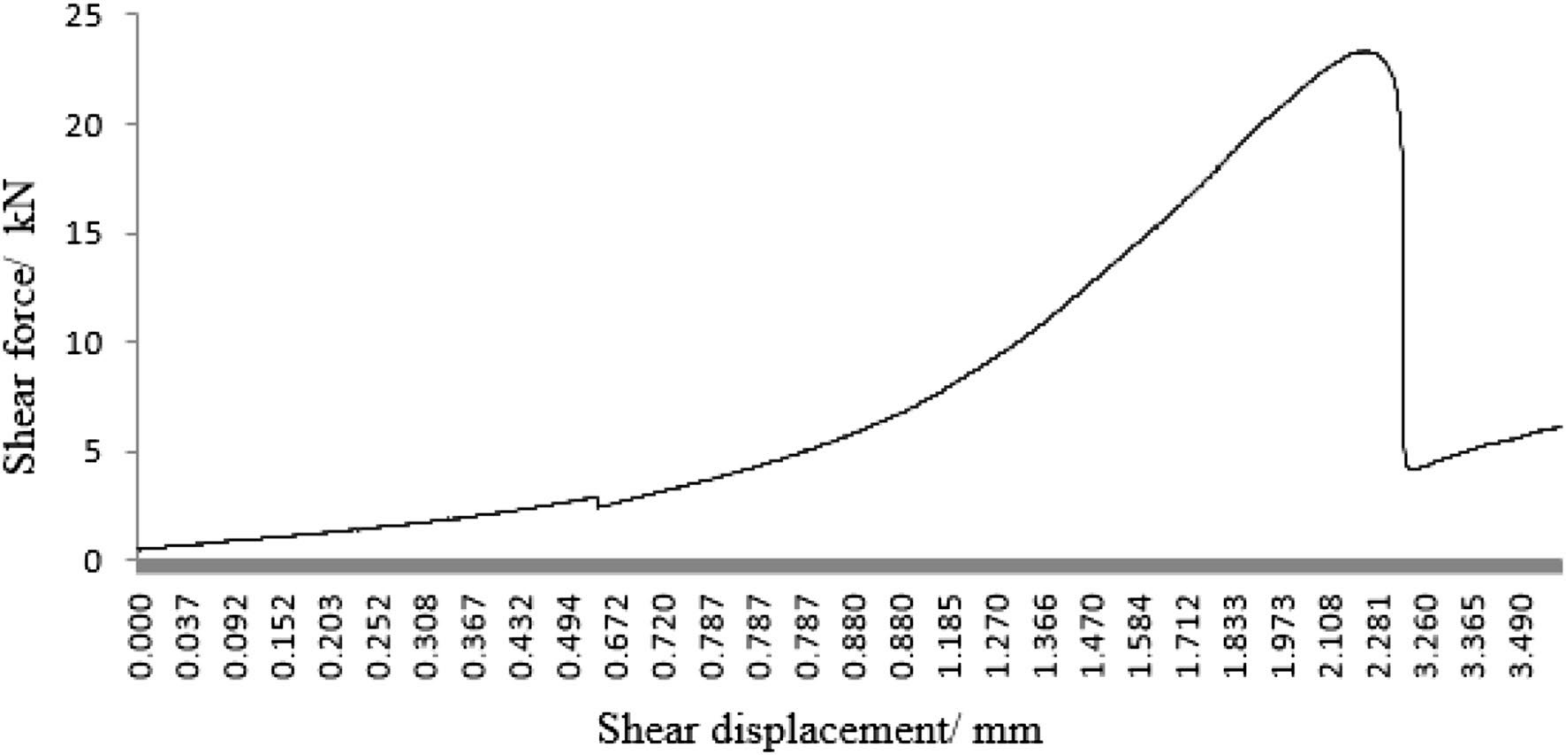
Shear force–shear displacement curve for a specimen containing 0–2.56 mm sand particles (produced from three grain size ranges).

**Figure 48:**
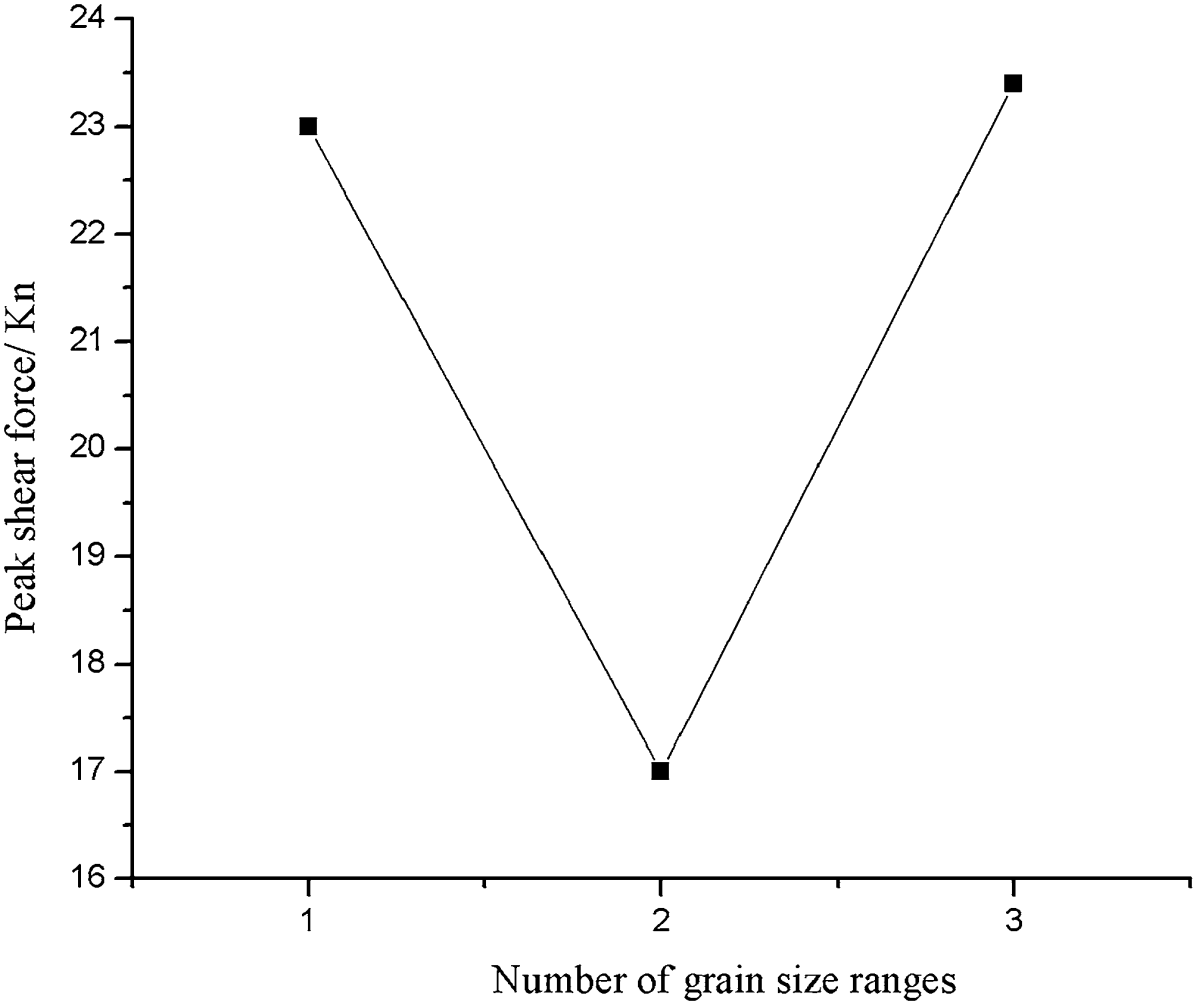
Peak shear stress–number of grain size range.

**Figure 49:**
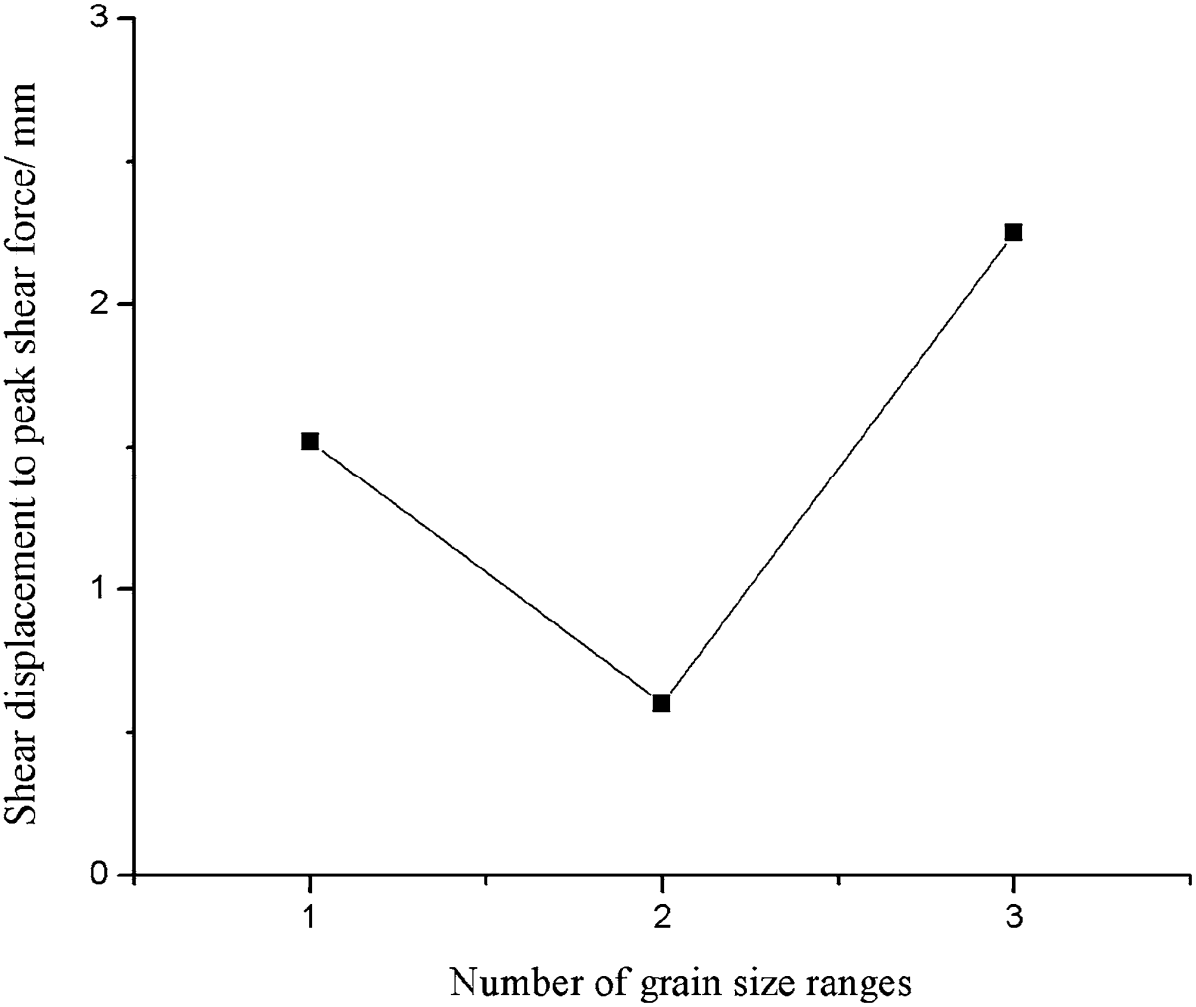
Shear displacement corresponding to the peak shear force–number of grain size range.

As can be seen from Figs. 45, 46, 47, 48 and 49, the peak shear stress for specimens consisting of three grain size ranges was greater than the peak shear stress for specimens consisting of two grain size ranges. The peak shear stress of the specimens consisting of three particle size ranges was the largest. As can be seen from Fig. 49, the shear displacement corresponding to the peak shear force for the three particle size ranges was greater than the shear displacement corresponding to the peak shear force for the two particle size ranges. The peak shear force corresponding to the shear displacement of the specimens consisting of three particle size ranges was the largest.

#### Grain size composition on acoustic emission characteristics

In order to investigate the effect of sand particle size on the acoustic emission characteristics of the specimens, specimens with the same fracture pattern were made.

**Figure 50:**
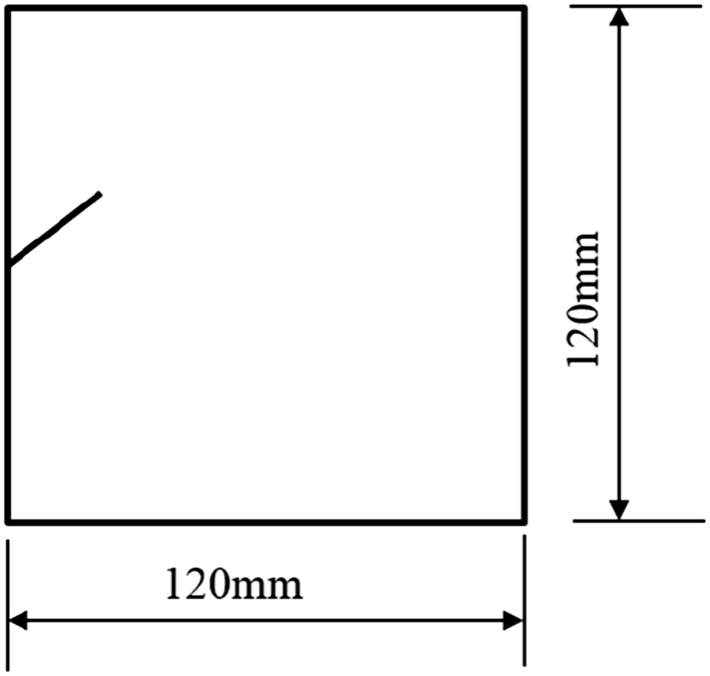
Fissure length 2 cm, horizontal angle of fissure 40° in specimens with different grain size ranges.

However, the specimens were made from sand of different particle size ranges. The first specimen contained sand in the particle size range of 1–2.56 mm. The second specimen contained equal amounts of sand in the 0–0.6 mm range and in the 1–2.56 mm range. The third specimen contained sand from all three grain size ranges. Thus containing sand from the entire 0–2.56 mm grain size range. The pattern of prefabricated cracks for these three specimens was shown in Fig. 50.

1. Acoustic emission characteristics in a specimen produced from a cement mortar mix containing sand in the 1–2.56 mm grain size range.

**Figure 51:**
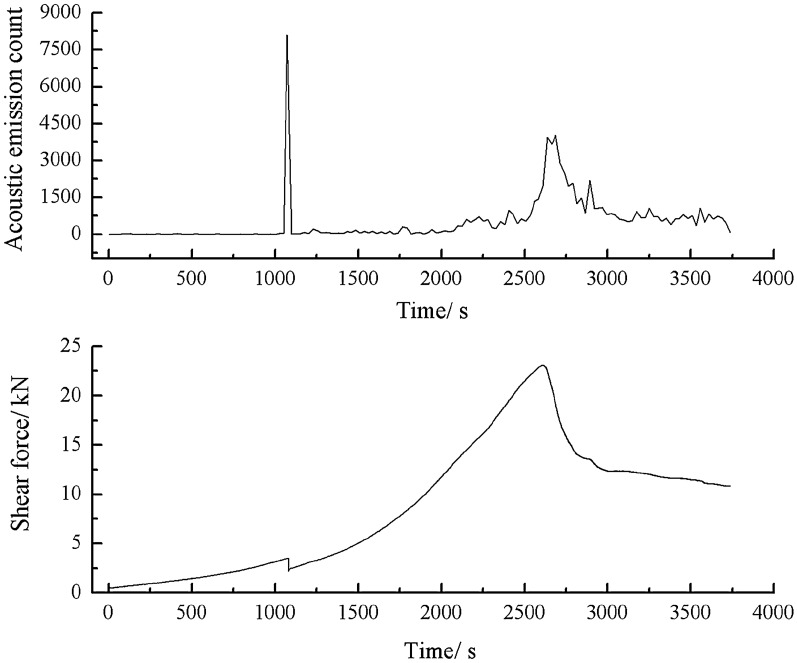
Shear stress–time and count-time curves for a specimen containing 1–2.56 mm sand particles (one grain size range).

**Figure 52:**
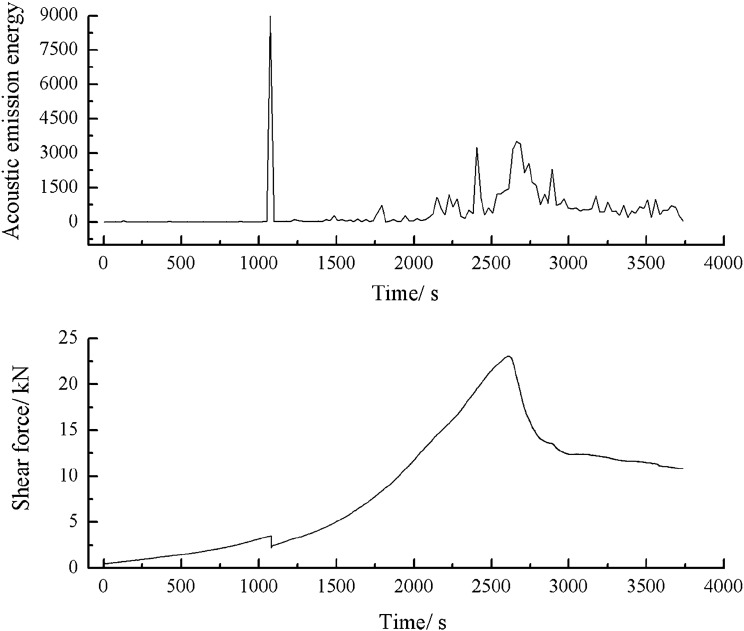
Shear stress–time and energy-time curves for a specimen containing 1–2.56 mm sand particles (one grain size range).

**Figure 53:**
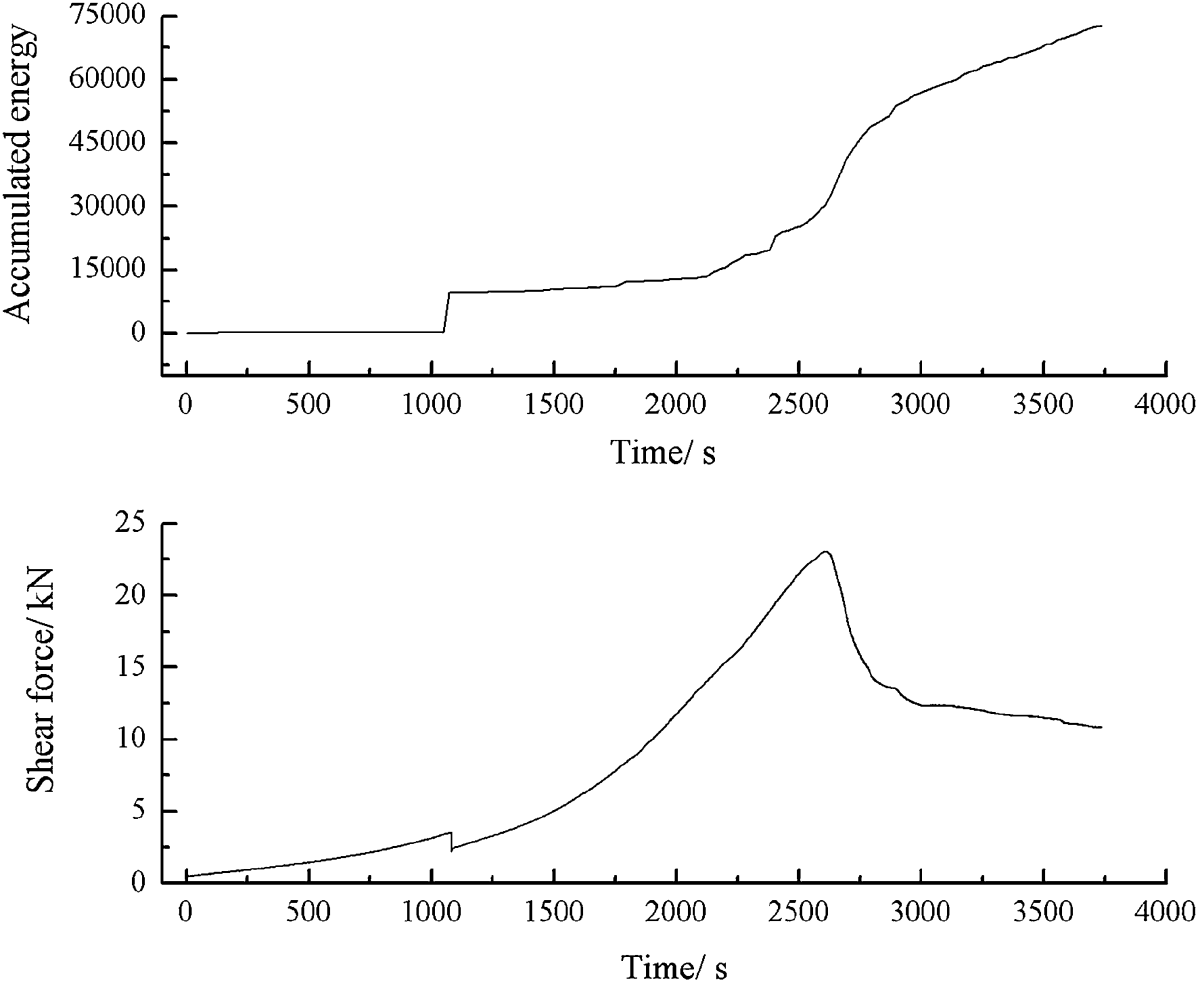
Shear stress–time and cumulative energy-time curves for a specimen containing 1–2.56 mm sand particles (one grain size range).

As can be seen from Figs. 51, 52 and 53, a specimen consisting of a range of grain sizes cracked at 1083 s, when there was a strong acoustic emission signal. The shear stress steadily increased before reaching its peak. However, small cracks were produced in the specimen, resulting in a small increase in energy and counts.

When the shear stress exceeded its peak, macroscopic cracks formed. The shear stress dropped sharply. Both the acoustic emission energy and counts rose rapidly. Finally, the specimen entered the residual strength phase, at which point the acoustic emission energy and counts were characterised by jumps.

2. Acoustic emission characteristics in a specimen produced from a cement mortar mix containing sand in the 0–0.6 mm and 1–2.56 mm grain size ranges.

**Figure 54:**
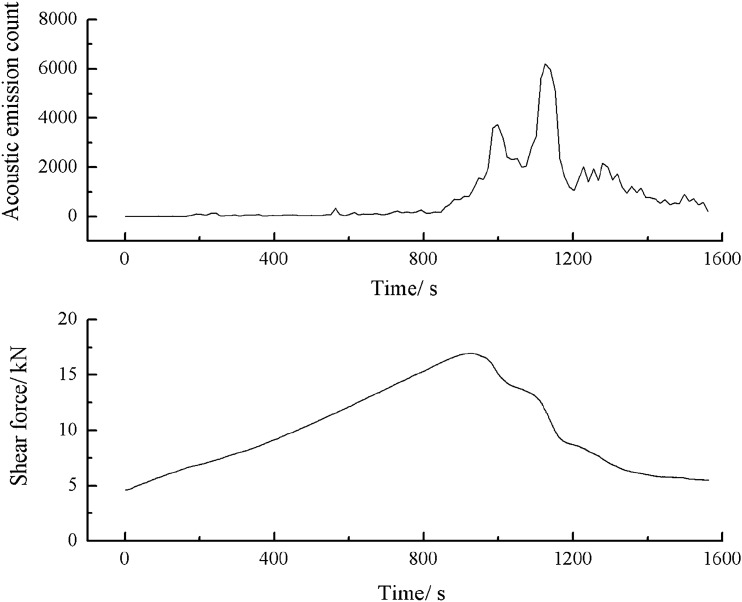
Shear stress–time and count-time curves for a specimen containing 0–0.6 mm and 1–2.56 mm sand particles (two grain size ranges).

**Figure 55:**
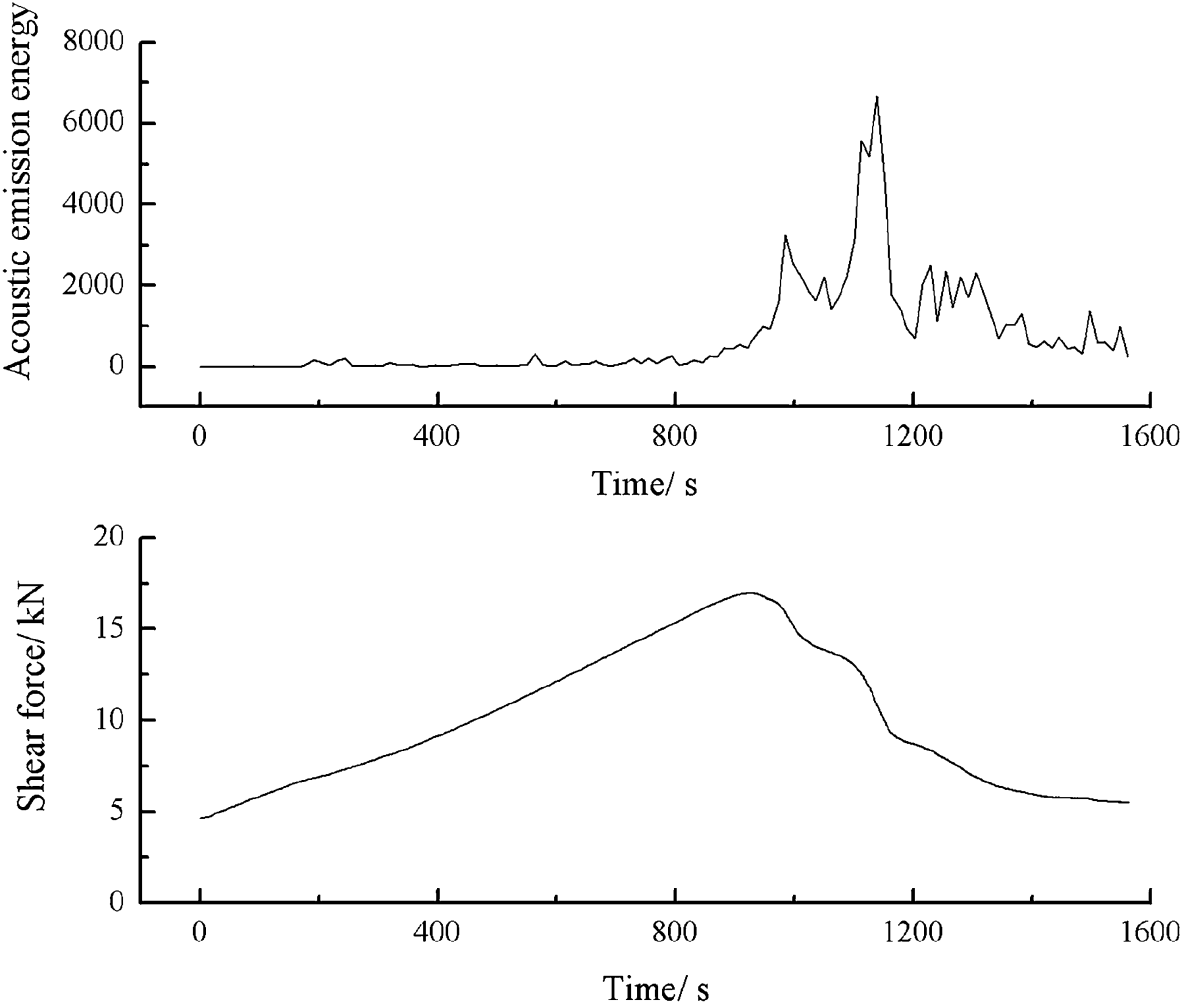
Shear stress–time and energy-time curves for a specimen containing 0–0.6 mm and 1–2.56 mm sand particles (two grain size ranges).

**Figure 56:**
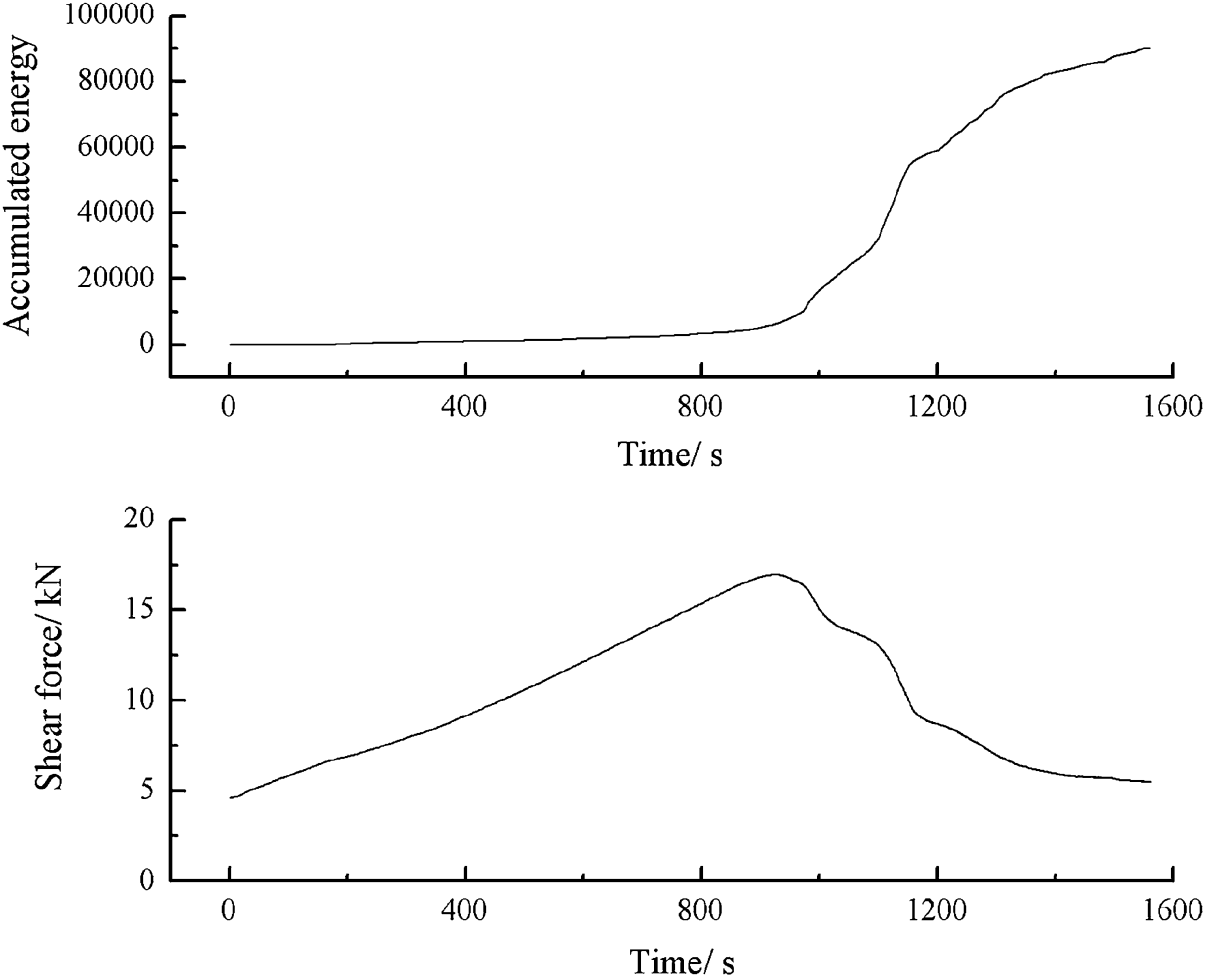
Shear stress–time and acumulative energy-time curves for a specimen containing 0–0.6 mm and 1–2.56 mm sand particles (two grain size ranges).

As can be seen from Figs. 54, 55 and 56, specimens consisting of both grain size ranges did not produce cracks until the shear force reached its peak. When the shear stress exceeded its peak, large cracks were produced in the specimens. The acoustic emission energy and counts rose rapidly. Thereafter, the specimens again produced large cracks. The acoustic emission energy and counts again rose rapidly. Finally, the specimen entered the residual strength stage. At this point the acoustic emission energy and counts increased by jumps.

3. Acoustic emission characteristics in a specimen produced from a cement mortar mix containing sand in the 0–2.56 mm grain size range.

**Figure 57:**
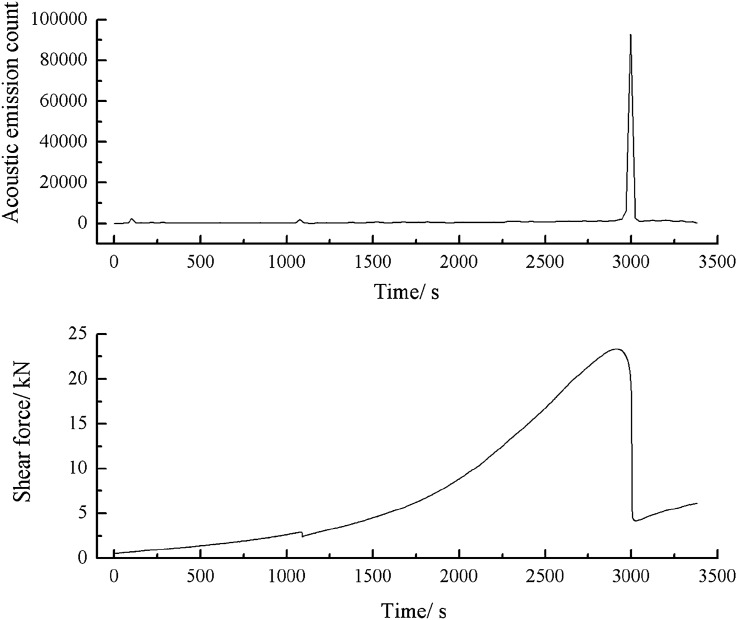
Shear stress–time and count-time curves for a specimen containing 0–2.56 mm sand particles (produced from three grain size ranges).

**Figure 58:**
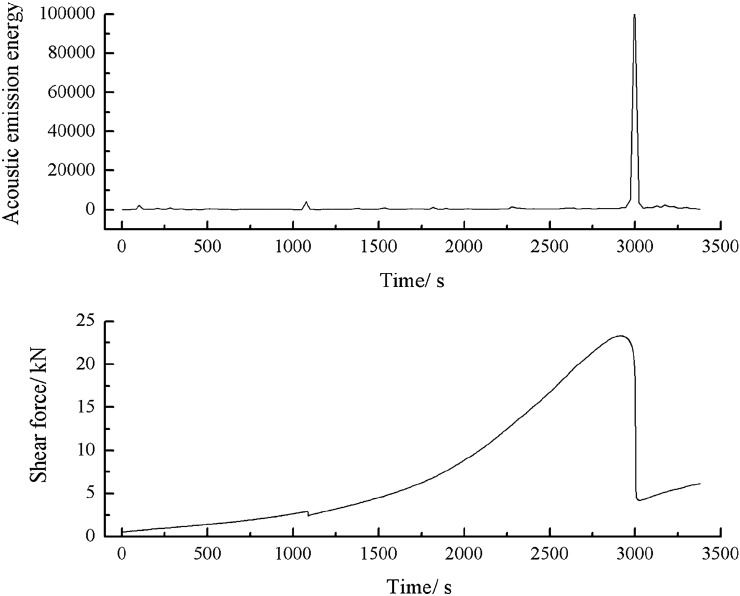
Shear stress–time and energy-time curves for a specimen containing 0–2.56 mm sand particles (produced from three grain size ranges).

**Figure 59:**
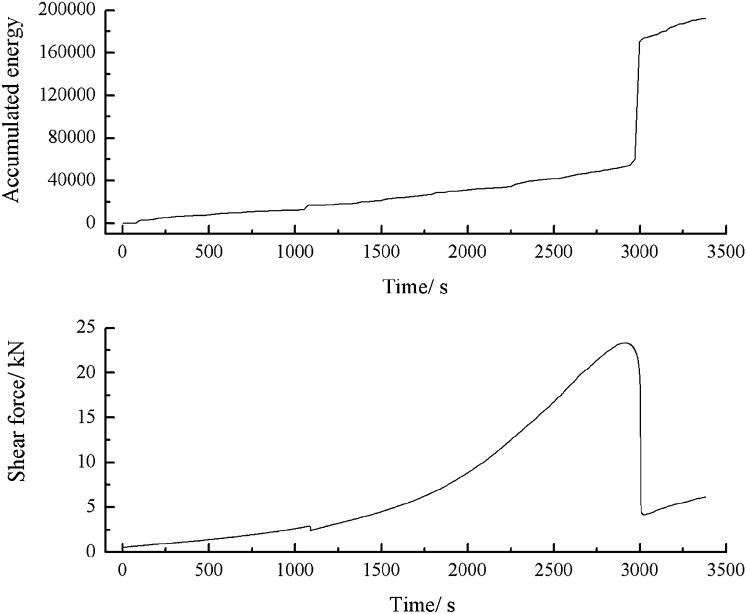
Shear stress–time and cumulative energy-time curves for a specimen containing 0–2.56 mm sand particles (produced from three grain size ranges).

As can be seen from Figs. 57, 58 and 59, specimens consisting of the three grain size ranges did not produce cracks until the shear stress reached its peak. After the peak shear stress was reached, the specimens developed large cracks. The acoustic emission energy and counts increased rapidly. The specimen was brittle fractured. Thereafter, the acoustic emission energy and metering were weak.

As can be seen from Figs. 53, 56 and 59, the cumulative energy of shear acoustic emission was the highest for a rock mass consisting of three grain size ranges. The cumulative shear acoustic emission energy of a rock mass consisting of one grain size range was the lowest.

The acoustic emission energy was the amount of energy remaining in the rock mass after the elastic strain energy contained in the rock mass has been removed from the energy required for the destruction of the rock mass and monitored by the acoustic emission device.

From the experiments, it was clear that the three grain size ranges comprised the rock masses with the highest peak shear and the most energy required for rupture. The three particle size ranges formed a rock mass because the gradation was better. The rock mass was also the strongest and most difficult to fracture because the grains were more fully embedded in each other. The rock mass also required the most energy to fracture. The rock mass was the most violent to fracture. The rock mass also had the highest acoustic emission energy.

### Fracture penetration patterns

**Figure 60:**
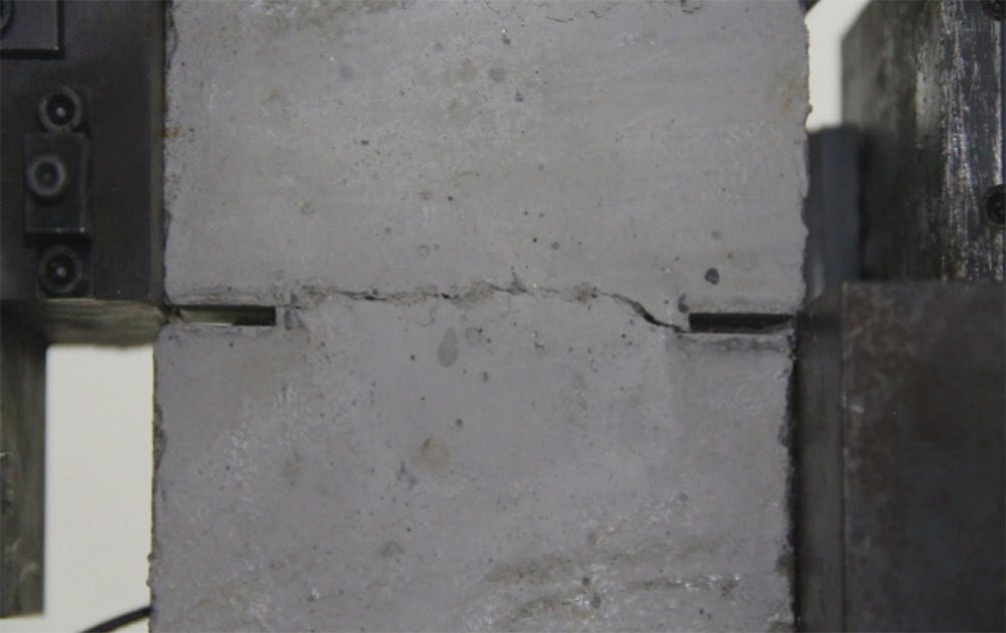
Photograph showing penetration patterns of the tip of the left crack and the tip of the right crack are directly connected.

**Figure 61:**
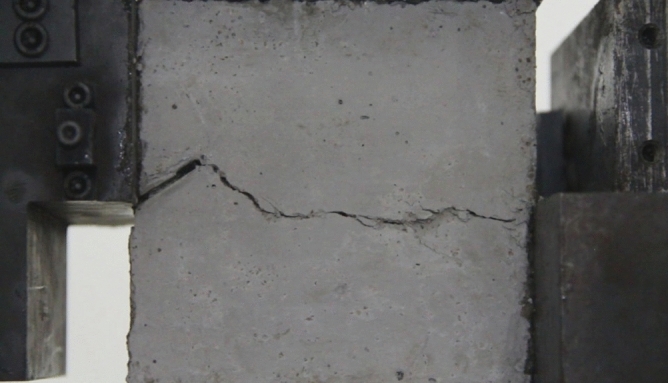
Photograph showing penetration patterns of the tip of the left crack and the right boundary are connected.

**Figure 62:**
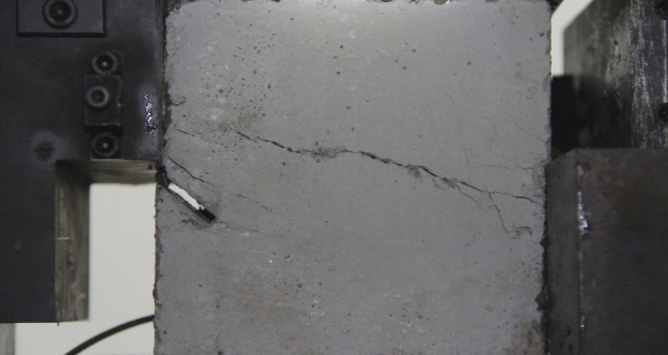
Photograph showing penetration patterns of the left boundary and the right boundary are connected.

**Figure 63:**
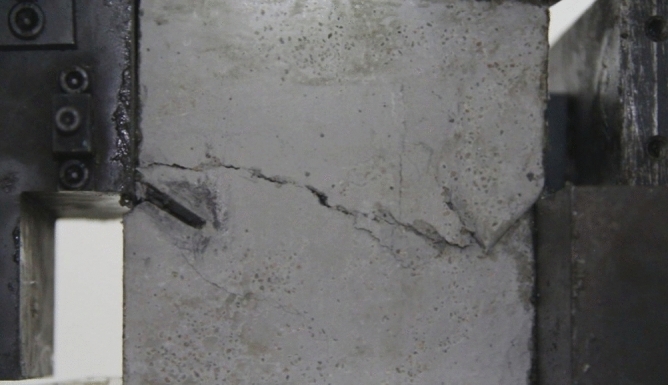
Photograph showing penetration patterns of the left boundary and the tip of the right crack are connected.

**Figure 64:**
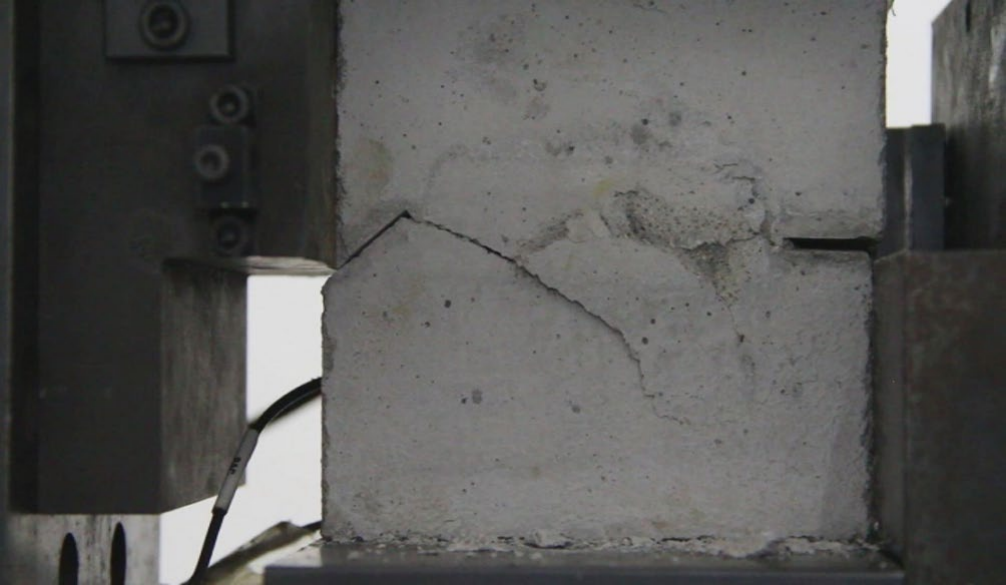
Photograph showing penetration patterns of the tip of the left crack and the tip of the right crack indirectly expand and connected.

From the tests of different specimens, the main modes of penetration of fracturing specimens are as follows:

As can be seen from Figs. 60, 61, 62, 63 and 64, the following types of crack penetration patterns can be identified.

1. The tip of the left crack and the tip of the right crack are directly connected and penetrated.
2. The tip of the left crack and the right boundary are connected and penetrated.
3. The left boundary and the right boundary are connected and penetrated.
4. The left boundary and the tip of the right crack are connected and penetrated.
5. The tip of the left crack and the tip of the right crack indirectly extended and connected.

## Conclusion

In this paper, the fracture patterns, mechanical properties and acoustic emission characteristics of rock masses made of rock-like materials with different prefabricated fracture patterns and different grain size compositions are investigated in compression shear. The conclusions are as follows:

1. Prefabricated fractures have a significant effect on the fracture mode and mechanical characteristics of the specimens.
2. Specimens consisting of different grain size ranges have different crack initiation sites and different specimen fracture patterns.
3. Specimens consisting of three particle size ranges have the highest peak shear force. The shear displacement corresponding to the peak shear force of a specimen consisting of three grain size ranges is the largest.
4. Specimens consisting of one grain size range produce relatively strong acoustic emissions before the peak shear. After the formation of the primary crack, the acoustic emission is jump-like. Specimens consisting of two grain size ranges produce strong acoustic emission after the peak shear. The acoustic emission rises again and jumps before the specimen enters the residual strength phase. Specimens consisting of three grain size ranges show strong acoustic emission at the time of main crack formation, followed by weak acoustic emission.
5. The cumulative energy of shear acoustic emission was the highest for a rock mass consisting of three grain size ranges. The cumulative shear acoustic emission energy of a rock mass consisting of one grain size range was the lowest. The three particle size ranges formed a rock mass because the gradation was better. The rock mass was also the strongest and most difficult to fracture because the grains were more fully embedded in each other. The rock mass also required the most energy to fracture.
